# Genome-wide identification and analysis of the MADS-box gene family in bread wheat (*Triticum aestivum* L.)

**DOI:** 10.1371/journal.pone.0181443

**Published:** 2017-07-25

**Authors:** Jian Ma, Yujie Yang, Wei Luo, Congcong Yang, Puyang Ding, Yaxi Liu, Linyi Qiao, Zhijian Chang, Hongwei Geng, Penghao Wang, Qiantao Jiang, Jirui Wang, Guoyue Chen, Yuming Wei, Youliang Zheng, Xiujin Lan

**Affiliations:** 1 Triticeae Research Institute, Sichuan Agricultural University, Chengdu, Sichuan, China; 2 Shanxi Key Laboratory of Crop Genetics and Molecular Improvement, Institute of Crop Science, Shanxi Academy of Agricultural Sciences, Taiyuan, China; 3 College of Agronomy, Xinjiang Agriculture University, Urumqi, China; 4 School of Veterinary and Life Sciences, Murdoch University, Murdoch WA, Australia; Saint Mary's University, CANADA

## Abstract

The MADS-box genes encode transcription factors with key roles in plant growth and development. A comprehensive analysis of the MADS-box gene family in bread wheat (*Triticum aestivum*) has not yet been conducted, and our understanding of their roles in stress is rather limited. Here, we report the identification and characterization of the MADS-box gene family in wheat. A total of 180 MADS-box genes classified as 32 Mα, 5 Mγ, 5 Mδ, and 138 MIKC types were identified. Evolutionary analysis of the orthologs among *T*. *urartu*, *Aegilops tauschii* and wheat as well as homeologous sequences analysis among the three sub-genomes in wheat revealed that gene loss and chromosomal rearrangements occurred during and/or after the origin of bread wheat. Forty wheat MADS-box genes that were expressed throughout the investigated tissues and development stages were identified. The genes that were regulated in response to both abiotic stresses (i.e., phosphorus deficiency, drought, heat, and combined drought and heat) and biotic stresses (i.e., *Fusarium graminearum*, *Septoria tritici*, stripe rust and powdery mildew) were detected as well. A few notable MADS-box genes were specifically expressed in a single tissue and those showed relatively higher expression differences between the stress and control treatment. The expression patterns of considerable MADS-box genes differed from those of their orthologs in *Brachypodium*, rice, and *Arabidopsis*. Collectively, the present study provides new insights into the possible roles of MADS-box genes in response to stresses and will be valuable for further functional studies of important candidate MADS-box genes.

## Introduction

In eukaryotes, the MADS-box gene family encodes transcription factors that play important roles in numerous biological functions by encoding transcription factors [1]. MADS-box transcription factors are characterized by the presence of a DNA binding domain that is approximately 60 amino acids length, known as the MADS domain, located at the N-terminal region of the protein. In plants, one of the most significant features of the MADS-box gene family is its essential role in the ABCDE model of flowering [2]. Numerous studies have identified its vital function in the formation and growth of floral organs [3], anthesis time [4], ovule development [5] and the ripening of fruits and seeds [6]. MADS-box gene family members have also been reported to be involved in stress responses [7], including abiotic and biotic responses [8]. For instance, the expression of *TaMADS2* was up-regulated after being infected by stripe rust fungus in wheat [9], and some MADS-box genes may also be involved in response to high salt concentrations [8]. In addition, the MADS-box gene plays an important role in the development of roots and trichomes [10].

In animals, plants and fungi, the MADS-box gene family has been categorized into two main groups, i.e., type I and type II (MIKC) [10,11]. Type I has been further classified into M-type and N-type genes [12]. In addition to the MADS domain, type II genes contain three additional domains, i.e., an Intervening domain, Keratin-like domain and C-terminal domain. Type II genes can be divided into MIKC^C^-type and MIKC^*^-type genes based on the structural divergence of the Intervening domain [13,14]. A Bayesian classification of the MADS-box proteins in *Arabidopsis* categorized the proteins into five distinct groups (Mα, Mβ, Mγ, Mδ, and MIKC) [6]. This classification scheme is adopted throughout the present study.

Given its important roles, the MADS-box gene family has been widely characterized in many plant species, including *Arabidopsis* [6], *Brachypodium* [8], rice [15], maize [16], sorghum [16], apple [17], poplar [14], cucumber [18] and soybean [19]. Common wheat (*Triticum aestivum* L, AABBDD genome, 2n = 6x = 42) is one of the most important cereal crops. To date, a genome-wide analysis of MADS-box gene family in wheat is yet to be published. The publication of the draft genome sequences of the common wheat cultivar ‘Chinese Spring’ [20] and its two progenitors, *T*. *urartu* (AA genome, 2n = 14) [21] and *Aegilops tauschii* (DD genome, 2n = 14) [22] enables an informed analysis of the distribution and expression of MADS-box genes and the evolutionary processes that formed polyploidy wheat on a genome wide scale [23,24]. In addition to their important roles in the growth and development of plants, the MADS-box genes have also been linked to biotic and abiotic stress responses [8,15,25]. However, an adequately systematic analysis of MADS-box genes in response to stresses has not yet been reported. In this study, the MADS-box genes throughout the wheat genome were first identified, classified, and physically mapped onto chromosomes. Subsequently, the gene structure, protein motifs and expression patterns of these MADS-box genes were analyzed.

## Materials and methods

### Identification of the MADS-box genes in wheat

Three methods were used to comprehensively identify the maximum number of MADS domain-containing sequences in wheat. The first method (henceforth Name Search) utilized searching for the MADS-box gene family members in the *T*. *aestivum* genome on Ensembl Plants (http://plants.ensembl.org/Triticum_aestivum/) by inputting the keyword ‘MADS’ (in Jun, 2016). The second method (HMM analysis) utilized the HMMER-3.1b2 software package (http://hmmer.janelia.org/) [26], which was used to build hidden Markov model profiles from full Pfam alignment files for the MADS-box gene family (PF00319; http://pfam.sanger.ac.uk). Resulting models were further employed to search the wheat protein database (V2.1) [20] and *T*. *aestivum* chromosome 3B RELEASE 1.0 [27] (http://wheat-urgi.versailles.inra.fr/) and identify potential MADS-box proteins (E-value ≤1 e-10, with manual inspection of sequences near to this threshold). In the third method (BlastP search), the MADS transcription factor database (PF00319) was used to BlastP search (E-value ≤ 1 e-5, percent identity ≥ 95%) the wheat protein database to obtain protein IDs of matching sequences. Unique non-redundant wheat MADS-box gene family members for the following analysis were identified by performing multiple sequence alignments using Clustal W [28] and removing redundant gene sequences. The MADS-box gene IDs of *A*. *tauschii* and *T*. *urartu* were retrieved by Name Search from the Ensembl Plants database (http://plants.ensembl.org/) in June 2016. The MADS-box coding sequences for *A*. *tauschii* and *T*. *urartu* were retrieved from the downloaded data (ftp://ftp.ensemblgenomes.org/pub/plants/release-31/fasta in July 2016.). As some of the MADS-box genes were previously identified and named [29], we here used ‘pTaMADS’ refer to the previous IDs listed in Table 1 and TaMADS refer to the present identified IDs.

**Table 1:** MADS-box gene family identified in wheat.

Name	Accession Number	Type	Length (bp)	# of Exons	# of Introns	Homologous wheat cDNA&
*TaMADS1*	*Traes_1AL_1B5F51626*.*1*	MIKC	—	7	6	AM502880/WPI1
*TaMADS2*	*Traes_1AL_5F5A87122*.*1*	MIKC	3194	7	6	AM502895
*TaMADS3*	*Traes_1AL_6B108514B*.*1*	MIKC	9223	5	4	AM502861/TaAGL7
*TaMADS4*	*Traes_1AL_6F5982F88*.1**	MIKC	396	—	—	
*TaMADS5*	*Traes_1AL_F3452F0E7*.*1*	Mα	2355	6	5	
*TaMADS6*	*Traes_1AS_24511D656*.*1*	MIKC	335	2	1	
*TaMADS7*	*Traes_1AS_3232D5589*.1**	MIKC	233	—	—	
*TaMADS8*	*Traes_1AS_985BB33A1*.*1*	MIKC	5784	6	5	
*TaMADS9*	*Traes_1BL_B44C0D37C*.*1*	MIKC	—	7	6	AM502894
*TaMADS10*	*Traes_1BL_F1D5BF5F8*.*1*	MIKC	9430	2	1	
*TaMADS11*	*Traes_1BS_05948C723*.1**	Mα	227	—	—	
*TaMADS12*	*Traes_1BS_1202C8C0D*.*1*	MIKC	728	2	1	
*TaMADS13*	*Traes_1BS_40F6DB3E3*.1*	MIKC	3570	7	6	
*TaMADS14*	*Traes_1BS_7366111C0*.1**	MIKC	230	—	—	
*TaMADS15*	*Traes_1BS_B03D4CD04*.*1*	MIKC	—	3	2	
*TaMADS16*	*Traes_1DL_0E4BA3B9A*.*1*	Mα	—	6	5	
*TaMADS17*	*Traes_1DL_6DA0DFC5B*.1**	MIKC	—	—	—	
*TaMADS18*	*Traes_1DL_81AB2AE99*.1**	Mα	—	—	—	
*TaMADS19*	*Traes_1DL_D25CDC57D*.1**	MIKC	1054	—	—	
*TaMADS20*	*Traes_1DL_D5BBCA2D0*.1**	MIKC	237	—	—	
*TaMADS21*	*Traes_1DS_A0312C264*.*1*	MIKC	459	2	1	
*TaMADS22*	*Traes_1DS_F22A3DB6A*.1*	MIKC	6433	8	7	AM502863/WAG
*TaMADS23*	*Traes_1DS_F70AAB507*.1**	MIKC	230	—	—	
*TaMADS24*	*Traes_2AL_0C169500B*.1**	MIKC	212	—	—	
*TaMADS25*	*Traes_2AL_20C2D79E1*.*1*	MIKC	6256	8	7	
*TaMADS26*	*Traes_2AL_267502097*.*1*	MIKC	—	2	1	AM502900
*TaMADS27*	*Traes_2AL_5820BAF68*.*1*	Mα	5191	7	6	
*TaMADS28*	*Traes_2AL_8DF89AA72*.*1*	MIKC	1824	6	5	
*TaMADS29*	*Traes_2AS_E2C631DBE*.*1*	MIKC	2201	7	6	
*TaMADS30*	*Traes_2AS_F79B671A6*.1**	MIKC	—	—	—	
*TaMADS31*	*Traes_2BL_26F24E716*.*1*	MIKC	6516	8	7	AM502871/TaAGL29
*TaMADS32*	*Traes_2BL_3E613DE21*.1*	MIKC	820	2	1	
*TaMADS33*	*Traes_2BL_50D716999*.*1*	Mα	2023	5	4	
*TaMADS34*	*Traes_2BL_ABBC40952*.1**	MIKC	212	—	—	
*TaMADS35*	*Traes_2BL_E0978B1BC*.1**	MIKC	1216	—	—	
*TaMADS36*	*Traes_2BS_4818EA1FF*.1*	MIKC	18641	7	6	AM502870/TaAGL10
*TaMADS37*	*Traes_2DL_3C9A3DD05*.1**	MIKC	193	—	—	
*TaMADS38*	*Traes_2DL_662837152*.1*	MIKC	977	3	2	
*TaMADS39*	*Traes_2DL_6A10DD109*.1**	MIKC	—	—	—	
*TaMADS40*	*Traes_2DL_6CD5A5CD9*.1*	MIKC	1802	6	5	
*TaMADS41*	*Traes_2DL_71F120931*.1*	MIKC	1472	6	5	
*TaMADS42*	*Traes_2DL_903A29CBA*.*1*	MIKC	6354	8	7	
*TaMADS43*	*Traes_2DS_4F6BA4A13*.*1*	MIKC	2241	7	6	
*TaMADS44*	*Traes_2DS_F20630B9F*.1**	MIKC	188	—	—	
*TaMADS45*	*Traes_3AL_01EE581F9*.*1*	MIKC	—	2	1	
*TaMADS46*	*Traes_3AL_219064574*.*1*	MIKC	1955	6	5	
*TaMADS47*	*Traes_3AL_4C4A0BCD8*.1**	Mα	1670	—	—	
*TaMADS48*	*Traes_3AL_8A8A03FF6*.1**	Mα	—	—	—	
*TaMADS49*	*Traes_3AL_B60854C6F*.*1*	Mα	681	2	1	
*TaMADS50*	*Traes_3AS_55E9080C2*.1*	MIKC	6639	7	6	AM502898/TaAGL39
*TaMADS51*	*Traes_3AS_B4247C855*.1**	MIKC	—	—	—	
*TaMADS52*	*Traes_3B_5C87F790C*.*1*	Mα	3445	6	5	
*TaMADS53*	*Traes_3B_7B4A1B60E*.*1*	Mα	—	1	0	
*TaMADS54*	*Traes_3B_87AC5133F*.1**	MIKC	231	—	—	
*TaMADS55*	*Traes_3B_D3F189425*.1**	Mα	131	—	—	
*TaMADS56*	*Traes_3B_E91A0554B*.*1*	Mα	681	2	1	
*TaMADS57*	*TRAES3BF001600030CFD_c1*	Mα	4870	3	2	
*TaMADS58*	*TRAES3BF009000010CFD_c1*	Mα	30589	1	0	
*TaMADS59*	*TRAES3BF009000040CFD_c1*	Mα	31104	1	0	
*TaMADS60*	*TRAES3BF021600020CFD_c1*	MIKC	2189	6	5	AM502881/WPI2
*TaMADS61*	*TRAES3BF024100070CFD_c1*	Mγ	1052	1	0	
*TaMADS62*	*TRAES3BF048900050CFD_c1*	MIKC	1924	6	5	AM502882/TaAGL14
*TaMADS63*	TRAES3BF050900030CFD_c1**	MIKC	6350	—	—	
*TaMADS64*	*TRAES3BF068500020CFD_c1*	MIKC	1303	6	5	
*TaMADS65*	*TRAES3BF073700150CFD_c1*	Mγ	755	1	0	
*TaMADS66*	*TRAES3BF077200070CFD_c1*	Mγ	362	1	0	
*TaMADS67*	*TRAES3BF106100060CFD_c1*	Mα	782	1	0	
*TaMADS68*	*TRAES3BF154600030CFD_c1*	Mγ	587	1	0	
*TaMADS69*	*Traes_3DL_0DEA285BD*.*1*	MIKC	—	3	2	
*TaMADS70*	*Traes_3DL_FBB80151F*.*1*	Mα	1258	3	2	
*TaMADS71*	*Traes_3DS_51A589227*.1*	MIKC	—	2	1	
*TaMADS72*	*Traes_3DS_767DC2DEB*.*1*	Mα	925	4	3	
*TaMADS73*	*Traes_3DS_B03D4CD04*.*1*	MIKC	—	3	2	
*TaMADS74*	*Traes_4AL_72A03AD23*.1**	MIKC	—	—	—	
*TaMADS75*	*Traes_4AL_8DBE120BC*.*1*	Mδ	—	11	10	
*TaMADS76*	*Traes_4AS_9A9C84490*.*1*	MIKC	2492	7	6	
*TaMADS77*	*Traes_4AS_A39F67523*.1*	MIKC	697	4	3	
*TaMADS78*	*Traes_4AS_BA5BB8032*.1*	MIKC	—	4	3	AM502897/TaAGL13
*TaMADS79*	*Traes_4AS_BE7BCEFEC*.1**	MIKC	—	—	—	
*TaMADS80*	*Traes_4AS_E1E60C5E5*.1**	MIKC	11504	—	—	
*TaMADS81*	*Traes_4BL_1A59E90E2*.1**	Mα	395	—	—	
*TaMADS82*	*Traes_4BL_410DEBFD3*.1*	MIKC	5358	4	3	
*TaMADS83*	*Traes_4BL_9A17EA3B7*.1**	Mδ	209	—	—	
*TaMADS84*	*Traes_4BL_B075EFE84*.1**	MIKC	—	—	—	AM502888/TaAGL38
*TaMADS85*	*Traes_4BL_B8FFB0854*.1**	Mα	191	—	—	
*TaMADS86*	*Traes_4BS_59C6DEC88*.1**	MIKC	184	—	—	
*TaMADS87*	*Traes_4BS_8B4AFA7C2*.*1*	Mδ	2867	11	10	
*TaMADS88*	*Traes_4DL_008F8BBFA*.1**	MIKC	—	—	—	
*TaMADS89*	*Traes_4DL_541814EAE*.*1*	MIKC	2202	7	6	AM502866
*TaMADS90*	*Traes_4DL_5633C0561*.1**	MIKC	181	—	—	
*TaMADS91*	*Traes_4DL_67EFB6303*.1**	MIKC	—	—	—	
*TaMADS92*	*Traes_4DL_964466BEC*.1**	MIKC	—	—	—	
*TaMADS93*	*Traes_4DL_AC7C7ABF3*.1**	MIKC	—	—	—	
*TaMADS94*	*Traes_4DL_C4CB3D5AF*.*1*	Mα	—	5	4	
*TaMADS95*	*Traes_4DS_4C4EB1D21*.*1*	Mδ	2866	11	10	
*TaMADS96*	*Traes_4DS_A28BC582A*.*1*	Mδ	—	8	7	
*TaMADS97*	*Traes_5AL_01329A110*.*1*	MIKC	5426	7	6	
*TaMADS98*	*Traes_5AL_13E2DEC48*.*1*	MIKC	—	5	4	
*TaMADS99*	*Traes_5AL_21C395CA8*.1*	MIKC	1211	6	5	
*TaMADS100*	*Traes_5AL_EBF32FE10*.1*	MIKC	3441	6	5	
*TaMADS101*	*Traes_5AS_029A65B0A*.1*	MIKC	373	2	1	
*TaMADS102*	*Traes_5AS_6AB7546BF*.*1*	Mα	—	1	0	
*TaMADS103*	*Traes_5AS_7BFB385EF*.*1*	MIKC	4050	7	6	AM502865/TaAGL2
*TaMADS104*	*Traes_5AS_B03D4CD04*.*1*	MIKC	963	3	2	
*TaMADS105*	*Traes_5AS_E9E60BA43*.*1*	MIKC	307	2	1	
*TaMADS106*	*Traes_5BL_4CA71C036*.*1*	MIKC	5361	8	7	AM502884/TaAGL27
*TaMADS107*	*Traes_5BL_5D2D22E67*.*1*	MIKC	—	7	6	
*TaMADS108*	*Traes_5BL_89636D032*.*1*	MIKC	2729	2	1	
*TaMADS109*	*Traes_5BL_9627436AE*.*1*	MIKC	7277	8	7	AM502875/'pTaMADS1'
*TaMADS110*	*Traes_5BS_284476236*.1**	MIKC	—	—	—	
*TaMADS111*	*Traes_5BS_A19FD8E34*.1**	MIKC	170	—	—	
*TaMADS112*	*Traes_5BS_BC15EF87A*.1*	MIKC	—	2	1	
*TaMADS113*	*Traes_5DL_8C647BFE2*.*1*	MIKC	—	7	6	
*TaMADS114*	*Traes_5DL_9CC4EC839*.*1*	MIKC	7311	8	7	AM502869/VRN-A1
*TaMADS115*	*Traes_5DS_16243E52C*.*1*	MIKC	4088	7	6	AM502864/TaAGL9
*TaMADS116*	*Traes_5DS_3EBE121C7*.1*	MIKC	657	3	2	
*TaMADS117*	*Traes_5DS_866BAC69D*.1*	MIKC	4652	2	1	
*TaMADS118*	*Traes_5DS_B288EE729*.1**	MIKC	470	—	—	
*TaMADS119*	*Traes_6AL_1F7DAC5FA*.*1*	MIKC	6730	8	7	
*TaMADS120*	*Traes_6AL_A93C6F2FC*.1**	MIKC	5198	—	—	AM502889/TaAGL11
*TaMADS121*	*Traes_6AL_B5D4C3A49*.*1*	MIKC	2828	2	1	
*TaMADS122*	*Traes_6AS_57E50EE92*.1**	MIKC	—	—	—	
*TaMADS123*	*Traes_6AS_9AA76345D*.*1*	MIKC	1755	7	6	
*TaMADS124*	*Traes_6AS_B4E415658*.1**	MIKC	—	—	—	
*TaMADS125*	*Traes_6AS_D6ABA1D79*.*1*	MIKC	6358	6	5	AM502883/TaAGL18
*TaMADS126*	*Traes_6BL_7C6B17284*.*1*	MIKC	—	8	7	AM502874/'pTaMADS12'
*TaMADS127*	*Traes_6BL_E1793636C*.1*	MIKC	3763	7	6	
*TaMADS128*	*Traes_6BS_43B59D772*.*1*	MIKC	887	4	3	
*TaMADS129*	*Traes_6BS_5789476CB*.1*	MIKC	1951	6	5	AM502893
*TaMADS130*	*Traes_6BS_8F1EC63B9*.*1*	MIKC	2019	7	6	
*TaMADS131*	*Traes_6DL_5B1D4DBF5*.*1*	MIKC	6874	8	7	AM502872/TaAGL37
*TaMADS132*	*Traes_6DL_609A01BD5*.1**	Mα	536	—	—	
*TaMADS133*	*Traes_6DL_B03D4CD04*.*1*	MIKC	963	3	2	
*TaMADS134*	*Traes_6DL_D1C1DBD34*.1*	MIKC	7049	6	5	AM502905
*TaMADS135*	*Traes_6DL_D8C9E421C*.1*	MIKC	4853	7	6	
*TaMADS136*	*Traes_6DS_2BAD7A60A*.1*	Mα	287	1	0	
*TaMADS137*	*Traes_6DS_3FB5A7717*.*1*	Mα	542	1	0	
*TaMADS138*	*Traes_6DS_9BBDCC9F7*.1*	MIKC	2051	6	5	
*TaMADS139*	*Traes_6DS_D50A0F246*.*1*	MIKC	897	4	3	
*TaMADS140*	*Traes_7AL_1C76E543C*.*1*	MIKC	1278	5	4	
*TaMADS141*	*Traes_7AL_67921A952*.*1*	MIKC	1904	7	6	
*TaMADS142*	*Traes_7AS_0CD3B69E7*.1*	MIKC	—	2	1	AM502877/TaAGL6
*TaMADS143*	*Traes_7AS_360247894*.1**	MIKC	287	—	—	
*TaMADS144*	*Traes_7AS_376CD50EA*.1**	MIKC	446	—	—	
*TaMADS145*	*Traes_7AS_8123257BA*.*1*	MIKC	8575	5	4	AM502903/TaAGL6
*TaMADS146*	*Traes_7AS_ADA694FCE*.1**	MIKC	—	—	—	
*TaMADS147*	*Traes_7AS_B38997CC0*.*1*	MIKC	1004	3	2	
*TaMADS148*	*Traes_7AS_C25A349A9*.*1*	MIKC	—	8	7	
*TaMADS149*	*Traes_7AS_CA6E66D75*.*1*	MIKC	2421	6	5	AM502891/VRT-2
*TaMADS150*	*Traes_7AS_EFE436F1D*.1*	MIKC	—	1	0	
*TaMADS151*	*Traes_7AS_F568FCBF1*.1**	Mα	1225	—	—	
*TaMADS152*	*Traes_7BL_7F4124E70*.1**	MIKC	—	—	—	
*TaMADS153*	*Traes_7BL_9BCF391CF*.*1*	MIKC	1908	7	6	
*TaMADS154*	*Traes_7BL_F5B6736D0*.1**	MIKC	184	—	—	
*TaMADS155*	*Traes_7BL_F621D9B9E*.1**	MIKC	4543	—	—	
*TaMADS156*	*Traes_7BS_209950516*.1**	MIKC	203	—	—	
*TaMADS157*	*Traes_7BS_4D5DE99CC*.1**	Mα	1360	—	—	
*TaMADS158*	*Traes_7BS_592EC3AB1*.*1*	MIKC	533	1	0	
*TaMADS159*	*Traes_7BS_7C0E94DFE*.*1*	MIKC	—	7	6	
*TaMADS160*	*Traes_7BS_9D42F9BEA*.1*	MIKC	10769	4	3	
*TaMADS161*	*Traes_7BS_DC9822CEF*.1*	MIKC	379	2	1	
*TaMADS162*	*Traes_7BS_F4CFCDF52*.1*	MIKC	—	5	4	
*TaMADS163*	*Traes_7DL_15FC3C682*.1**	Mα	233	—	—	
*TaMADS164*	*Traes_7DL_303249AE4*.1*	Mγ	434	1	0	
*TaMADS165*	*Traes_7DL_A773A64E2*.1**	Mα	207	—	—	
*TaMADS166*	*Traes_7DL_CAF83263E*.*1*	MIKC	1786	7	6	AM502878/TaAGL32
*TaMADS167*	*Traes_7DL_DDCC09B24*.*1*	MIKC	6019	7	6	AM502879/' pTaMADS51'
*TaMADS168*	*Traes_7DS_05F0F0B64*.1*	MIKC	—	2	1	
*TaMADS169*	*Traes_7DS_366A869CF*.1**	MIKC	188	—	—	
*TaMADS170*	*Traes_7DS_4AF5C695F*.1*	MIKC	2872	3	2	
*TaMADS171*	*Traes_7DS_4DF7EB08F*.1**	Mα	—	—	—	
*TaMADS172*	*Traes_7DS_59CBF5647*.1*	MIKC	371	1	0	
*TaMADS173*	*Traes_7DS_68DE33D2A*.*1*	MIKC	6378	8	7	AM502886/TaAGL8
*TaMADS174*	*Traes_7DS_7A30E1397*.1*	MIKC	—	5	4	
*TaMADS175*	*Traes_7DS_7D07B9FFA*.*1*	Mα	1693	5	4	
*TaMADS176*	*Traes_7DS_7F8C88C92*.1*	MIKC	—	1	0	
*TaMADS177*	*Traes_7DS_90668ED2B*.1*	MIKC	—	6	5	AM502892/VRT-2
*TaMADS178*	*Traes_7DS_C8938031B*.1**	MIKC	1816	—	—	
*TaMADS179*	*Traes_7DS_D435DF316*.1*	MIKC	184	1	0	
*TaMADS180*	*Traes_7DS_D9008CC09*.1*	MIKC	379	2	1	

* The full coding sequences of 38 MADS-box genes were deduced based on alignments between the retrieved gene models and the genome sequences.

** Fifty-six MADS-box genes do not have complete coding sequences.

The remaining 86 retrieved genes have full-length coding sequences.

The accession numbers were from Paolacci et al. [38].

### Classification of the MADS-box gene family

*Arabidopsis* genomes contain a rather ancient diversity of MADS-box genes that are representative of other flowering plants [30]. A total of 108 *Arabidopsis* MADS-box genes [6] (S1 Table) were selected for the purpose of classifying the types of MADS-box genes in wheat. Specifically, the obtained MADS-box protein sequences of wheat were aligned to those of *A*. *thaliana* [6] using Clustal X (http://www.clustal.org/) [31]. The alignment results were used to construct a phylogenetic tree using the neighbor-joining method with 1, 000 bootstrap replicates [32].The phylogenetic tree was visualized with the online software tool EvolView (http://www.evolgenius.info/) [31]. The wheat genes most similar to *Arabidopsis* MADS-box genes were considered to be the *Arabidopsis* orthologs. A phylogenetic tree used to infer the relationships among the identified MADS-box genes was also constructed by coding sequences using the neighbor-joining method. Manual inspection of the alignments were further conducted for several genes including T*raes_4AS_BE7BCEFEC*.*1* and *Traes_4BS_59C6DEC88*.*1*, in order to determine their relationships.

### Gene structure and conserved motif analysis

Coding sequences of the MADS-box genes with complete open reading frames were selected for phylogenetic analyses using Clustal X (http://www.clustal.org/) via the neighbor-joining method [33]. The genes that grouped together within the gene tree but were derived from different wheat sub-genomes were regarded as homeologous sequences of a same MADS-box gene [23]. The coding sequence of each gene was aligned with its genomic sequence to construct an exon/intron map in the Gene Structure Display Server program (http://gsds.cbi.pku.edu.cn/) [34]. Motifs of the MADS-box protein sequences were searched by using the online software MEME 4.11.3 (http://meme-suite.org/tools/meme) with the following parameters: number of repetitions—any, maximum number of motifs—20, optimum motif width set to≥ 6 and ≤ 200 [15]. The motifs obtained were annotated using the SMART and NCBI-SMARTBLAST search programs.

### Mapping MADS-box genes onto chromosomes

The daft physical genome sequences of wheat were downloaded from Ensembl Plants (ftp://ftp.ensemblgenomes.org/pub/plants/release-31/fasta/triticum_aestivum/) on August 27, 2016. All the identified MADS-box genes were BLASTed (E-value ≤ 1 e-5, percent identity ≥ 95%) against the genome sequences of the corresponding wheat chromosomes to determine their chromosomal locations. The MapInspect tool (http://mapinspect.software.informer.com/) was then used to draw their locations onto the physical map of each chromosome.

### Identification of the MADS-box orthologs in *T*. *urartu* and *A*. *tauschii*

The MADS-box genes of *A*. *tauschii* and *T*. *urartu* were retrieved from the collected coding sequences data downloaded from GIGA_DB (http://gigadb.org/) in 2013. Phylogenetic trees for the wheat–*T*. *urartu* and wheat–*A*. *tauschii* MADS-box gene pairs were constructed based on coding sequences in Clustal X using the neighbor-joining method with 1, 000 bootstrap replicates [32].Genes from different species that grouped together within each gene tree were designated as orthologs [35]. Based on these orthologous MADS-box genes, a collinear map of the *T*. *urartu*–wheat A genome and the *A*. *tauschii*–wheat D genome was created using genome visualization tool CIRCOS [36]. The locations of MADS-box orthologous genes on the chromosomes of *A*. *tauschii* and *T*. *urartu* were retrieved from the database published by Jia et al. [22] and Ling et al. [21], respectively.

### Expression analyses

The expression patterns throughout all the available tissues and developmental stages as well as gene expression responses to biotic and abiotic stresses for the identified MADS-box genes were analyzed. These data were retrieved from the expVIP Wheat Expression Brower (http://www.wheat-expression.com/) [37] as processed expression values in transcripts per million (TPM) obtained via RNA-seq analysis. The biotic stresses included inoculations with *Fusarium graminearum*, *Septoria tritici*, stripe rust, and powdery mildew pathogen. The abiotic stresses included phosphorus deficiency, drought, heat and combined drought and heat treatments. The details of the analyzed materials and treatments are presented in S2 Table. To determine the regulation patterns of a given gene subjected to a stress, the ratio of the expression value under a treatment to the control was calculated. Ratios under a given treatment that were greater than or less than 1.0 indicated that gene expression was altered by the stress treatment, while a ratio equal to 1.0 indicated the gene expression was unaltered by that treatment. The MeV online software tool (http://www.tm4.org/mev.html) was used to generate heatmaps form the obtained expression values or ratios.

## Results

### Identification and classification of MADS-box family genes in wheat

**Fig 1:**
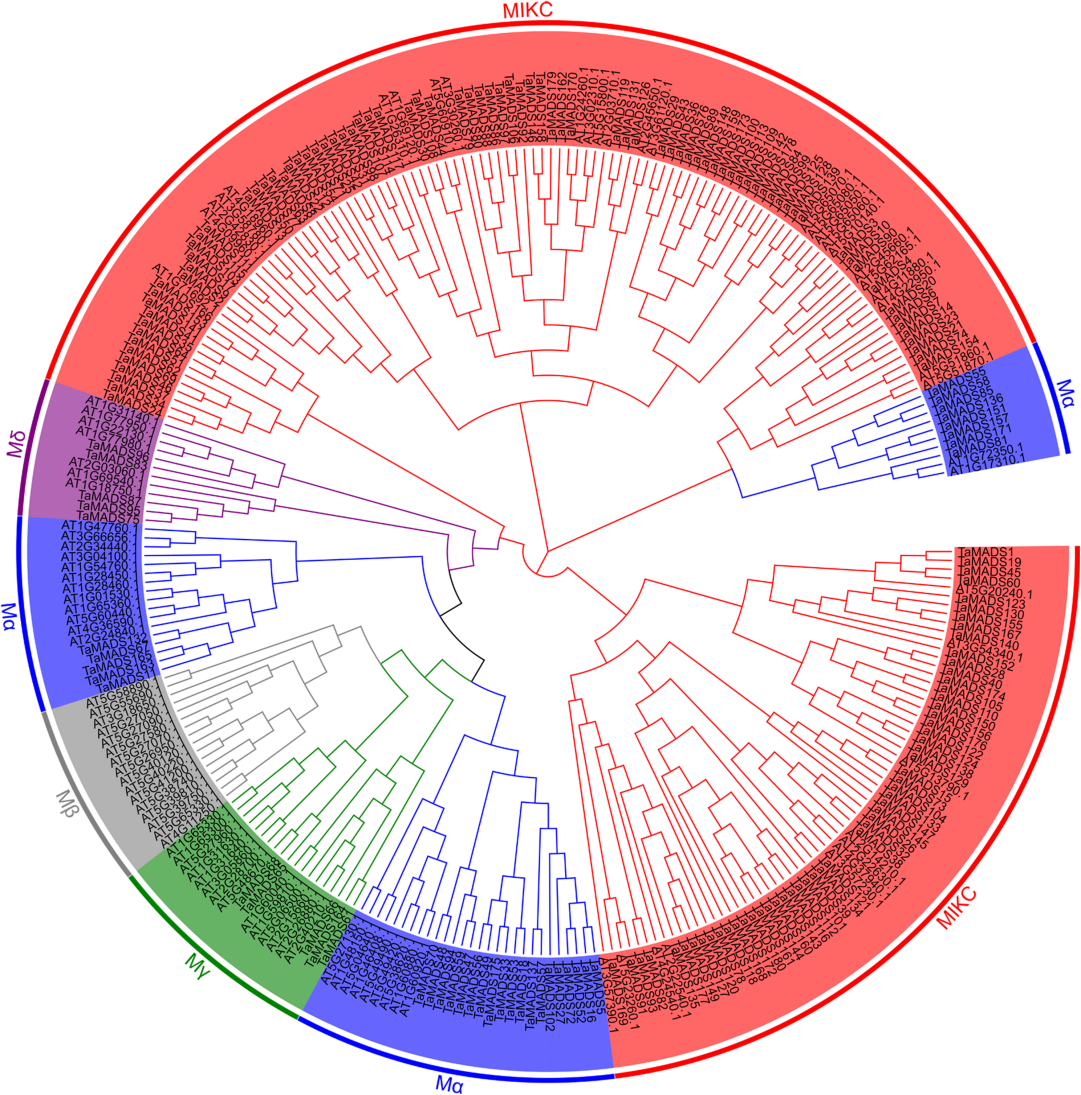
Phylogenetic relationship of MADS-box proteins between wheat and *Arabidopsis*. Five classes are represented by branches of different colors, including Mα (blue), Mβ (grey), Mγ (green), Mδ (purple), and MIKC (red).

In total, 142, 140 and 166 MADS-box genes were identified by Name Search, HMM analysis and BlastP search, respectively. Collectively, these methods identified 180 unique MADS-box genes for the subsequent analyses (Table 1). Reciprocal BlastN searches identified 32 sequences were homologous to MADS genes reported by Paolacci et al. [38]. Based on the classification scheme of MADS-box genes in *A*. *thaliana* (S1 Table), 32, 5, 5 and 138 wheat genes were identified to be Mα-, Mγ-, Mδ- and MIKC-type, MADS-box genes, respectively (Fig 1 and Table 1).

Furthermore, an un-rooted tree of the 180MADS-box genes was constructed (S1 Fig) to determine their phylogenetic relationships. All the sequences were divided into 101 groups. Of them, 25 groups representing 75 genes consisted of three genes from each of the different sub-genomes that were regarded as orthologous copies of a single MADS-box gene. Twenty-two of these twenty-five groups were on different chromosomes but were still from the same homeologous group (e.g., *Traes_1AL_5F5A87122*.*1*, *Traes_1BL_B44C0D37C*.*1* and *Traes_1DL_6DA0DFC5B*.*1*). For the remaining 3 groups among these 25 groups, two of the three genes were on different chromosomes (e.g., *Traes_7AS_CA6E66D75*.*1*, *Traes_7DS_90668ED2B*.*1* and *Traes_6BL_E1793636C*.*1*). Of the 29 groups each containing two genes, 3 were from a single chromosome (e.g., *Traes_4DS_A28BC582A*.*1* and *Traes_4DL_964466BEC*.*1*), 15 from the same homeologous group (e.g., *Traes_1AL_5F5A87122*.*1* and *Traes_1DL_D25CDC57D*.*1*), and 11 from different homeologous groups (e.g., *Traes_1BS_1202C8C0D*.*1* and *Traes_3DS_51A589227*.*1*). The remaining 47 groups consisted of only one gene (e.g., *Traes_4BL_1A59E90E2*.*1*). The MIKC-type genes were distributed on each of the 21 chromosomes. The Mα-type genes were detected on 15 of the 21 chromosomes. The Mδ- and Mγ-type genes were detected on three (4A, 4B and 4D) and two (3B and 7D) chromosomes only, respectively (Table 1).

### Gene and protein structures of the wheat MADS-box genes

**Fig 2:**
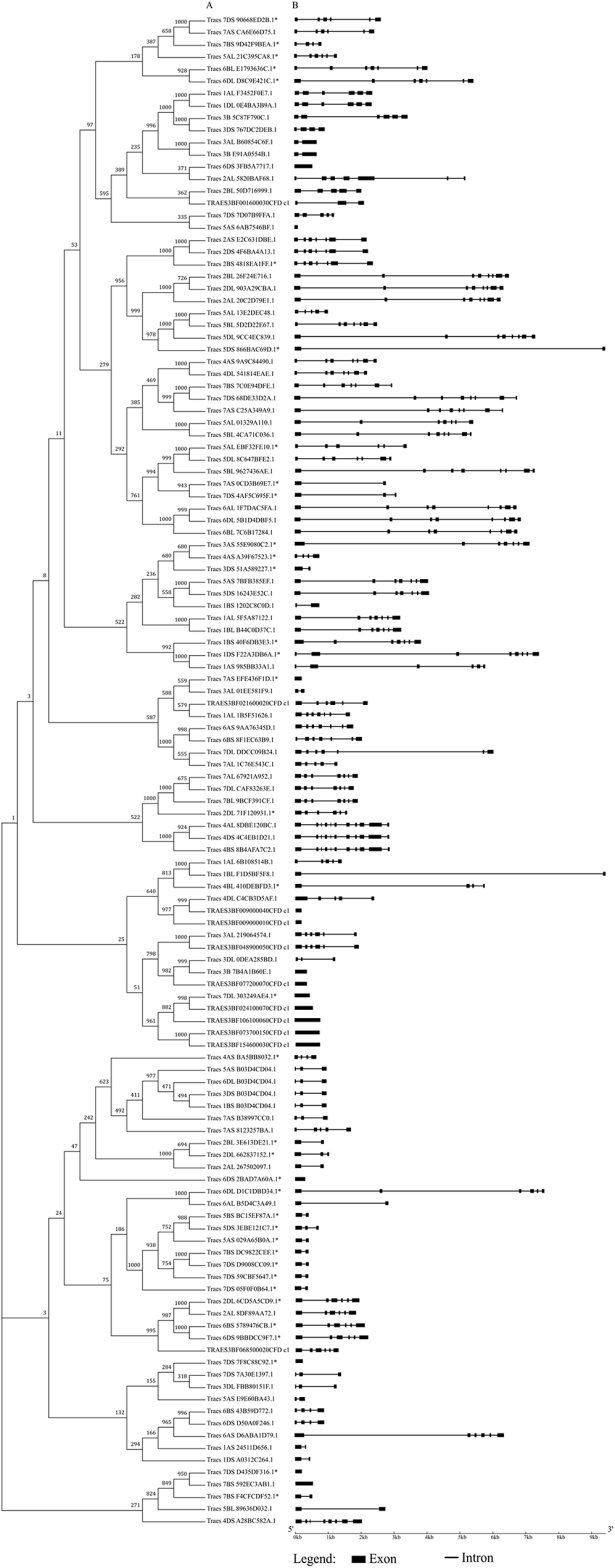
Gene structures of 124 wheat MADS-box genes with full-length coding sequences. The phylogenetic tree of MADS-box genes were constructed by the neighbor-joining method with 1000 bootstrap replicates. Lengths of exons and introns of each MADS-box gene were displayed proportionally. Exons are represented by black boxes and introns by black lines. The sizes of exons and introns can be estimated using the scale below. The full coding sequences of 38 MADS-box genes indicated by '*' were deduced based on alignments between the retrieved gene models and the genome sequences.

The phylogenetic tree of the MADS-box genes were based on 124 full-length coding sequences (Fig 2). The average lengths of the MADS-box genes varied among the different MADS types. For example, Mα-type genes were 3,704 bp in average length while Mγ-types genes were 639 bp in average length (Table 1). As expected, the distribution of introns in wheat was similar to those in in *Arabidopsis* [6] and rice [15]. Specifically, MIKC (four introns per gene) and Mδ genes (nine introns per gene) contained multiple introns and Mα (two introns per gene) and Mγ genes (zero intron per gene) usually had no intron or just one to two intron(s) (Fig 2). Closely related genes are generally more similar in gene structure, and the most obvious differences only exist in lengths of introns and exons. Additionally, some close gene pairs did differ in intron/exon arrangements. For example, *Traes_6AS_D6ABA1D79*.*1* had six exons, whereas its close homeologs *Traes_6BS_43B59D772*.*1* and *Traes_6DS_D50A0F246*.*1* both had four, although their phylogenetic relationship was supported by a nearly 90% bootstrap value (Fig 2).

**Fig 3:**
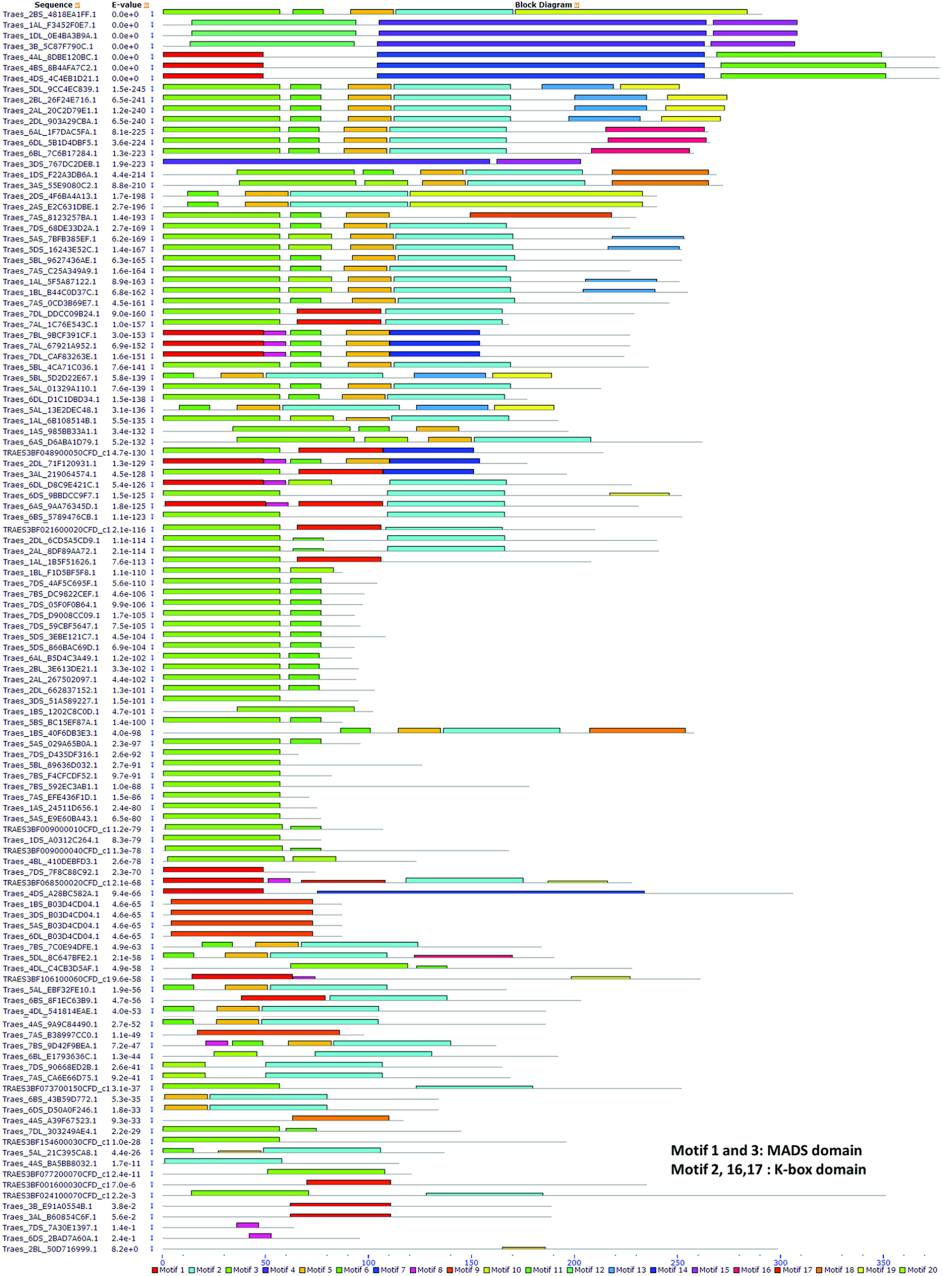
Protein motif of wheat MADS-box proteins. Each motif is represented by a number in a colored box. Details of motif were listed in S3 Table.

As shown in Fig 3, nearly all the wheat MADS proteins had either MADS or K-box domain motifs. Additionally, motifs 1 and 3 were localized within the MADS-box domain, while motifs 2, 16, and 17 (S3 Table) were in the K-box domain. In addition, unknown motifs were also identified by MEME motif analysis (S3 Table and Fig 3).

### Chromosomal locations of MADS-box gene family members in wheat

**Fig 4:**
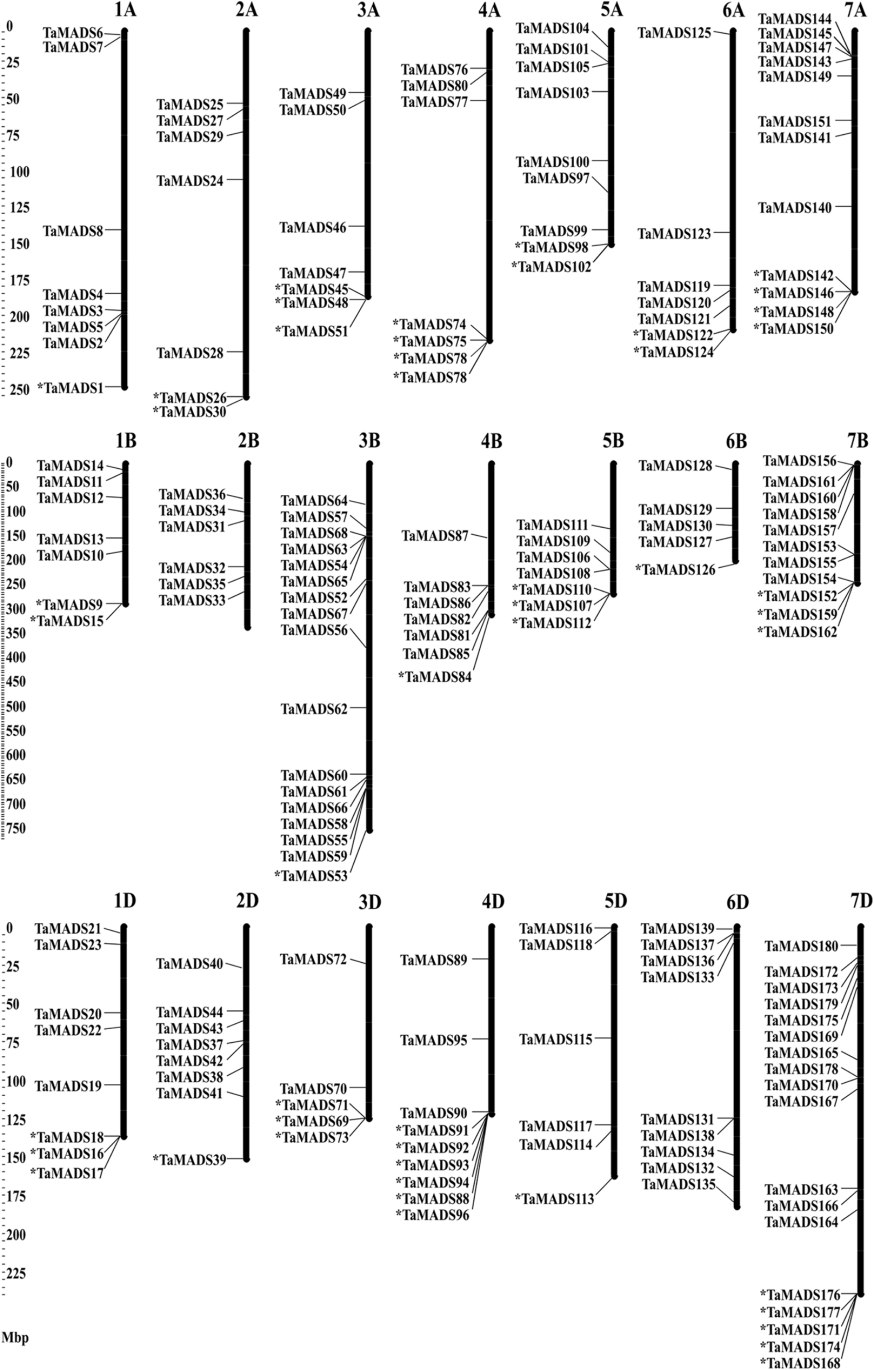
Chromosome distributions of MADS-box genes in wheat. The wheat MADS-box genes numbered from *TaMADS1* to *TaMADS180*. The locations of the genes represented by ‘*’ were not determined and were placed at the end of each corresponding chromosome.

Of the 180 MADS-box genes, the precise physical locations of 132 could be mapped onto chromosomes (Fig 4). As expected, each of the 180 genes was non-randomly distributed among A (57), B (60) and D (63) sub-genomes, respectively. This is also reflected among each of the seven homeologous MADS-box groups. Notably, bias changes in gene number were observed among the homeologous groups. The seventh homeologous group contained nearly twice the number of MADS-box genes (41) observed among the others with 11 for 7B, 12 for 7A and 18 for 7D, respectively. The MADS-box gene numbers ranging from 21 to 29 did not differ much among the remaining homeologous groups (Table 1 and Fig 4).

### Phylogenetic analysis of the *T*. *urartu*, *A*. *tauschii*, and wheat orthologs

**Fig 5:**
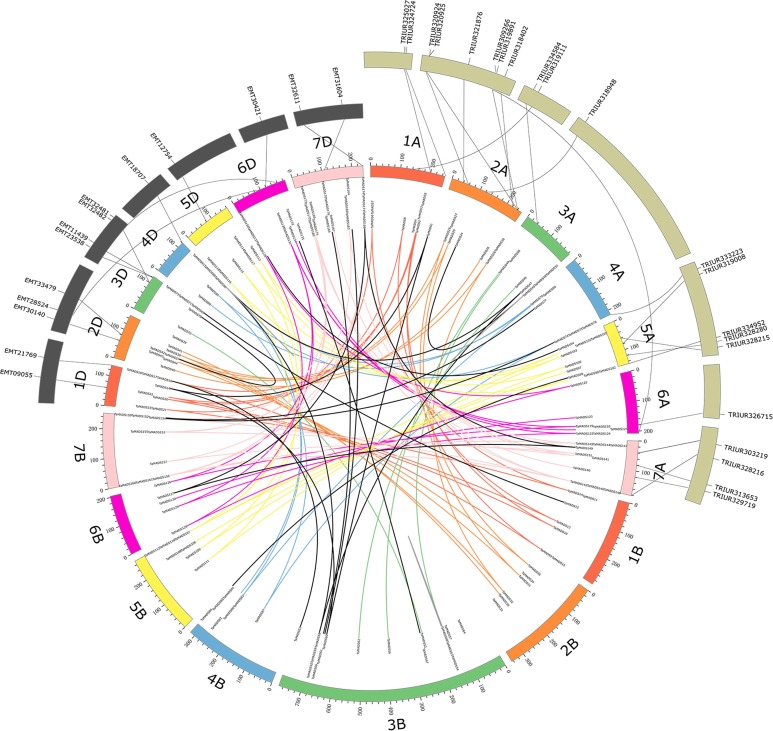
Collinear analysis for the MADS-box gene family among wheat, *T*. *urartu* and *A*. *tauschii*. The gray annulus on the top left represent chromosomes of *A*. *tauschii* and the olive annulus on the top right represent chromosomes of *T*. *urartu*. Seven homologous groups of wheat chromosomes are represented in different colors. Homeologous genes of each group are linked by lines with corresponding color. The collinearity was signified by the gray lines based on 21 and 14 pairs of orthologous genes in wheat and *T*. *urartu* as well as wheat and *A*. *tauschii*, respectively.

A total of 84 *T*. *urartu-*MADS, 57 *T*. *aestivum*-A-MADS, 97 *A*. *tauschii*-MADS, and 63 *T*. *aestivum*-D-MADS gene sequences were used to construct gene trees. Of the 33 pairs of *T*. *urartu*-wheat A genome orthologs (S2 Fig and S4 Table), 21 could be mapped to *T*. *urartu* chromosomes with 2 on 1A, 6 on 2A, 2 on 3A, 1 on 4A, 5 on 5A, 1 on 6A and 4 on 7A (Fig 5). Of the 32 pairs of *A*. *tauschii*-wheat D genome orthologs (S3 Fig and S4 Table), only 14 could be mapped to *A*. *tauschii* chromosomes with 2 on 1D, 3 on 2D, 4 on 3D, 1 on 4D, 1 on 5D, 1 on 6D, and 2 on 7D (Fig 5). Most of the orthologs (91% and 88% for *T*. *urartu* and *A*. *tauschii*, respectively,) belonged to MIKC-type MADS-box genes, as expected given this type’s high proportional composition (77%) among the previously identified wheat MADS-box genes (S4 Table).The chromosome locations of most wheat MADS-box genes and their orthologs in *T*. *urartu* and *A*. *tauschii* could be corresponded to each other (S4 Table). However, *TaMADS8*, *24*, *75* and *124* on wheat chromosomes 1AS, 2AL, 4AL and 6AS had corresponding orthologs on 3AL, 4AS, 5AL and 2AL, respectively in *T*. *urartu*. Another two genes, *TaMADS132* and *138* on wheat 6DL and 6DS had corresponding orthologs on 3D and 2D, respectively, in *A*. *tauschii* (Fig 5).

### Expression profiles of MADS-box genes in response to stresses, during vegetative and reproductive development

#### Abiotic stress

**Fig 6:**
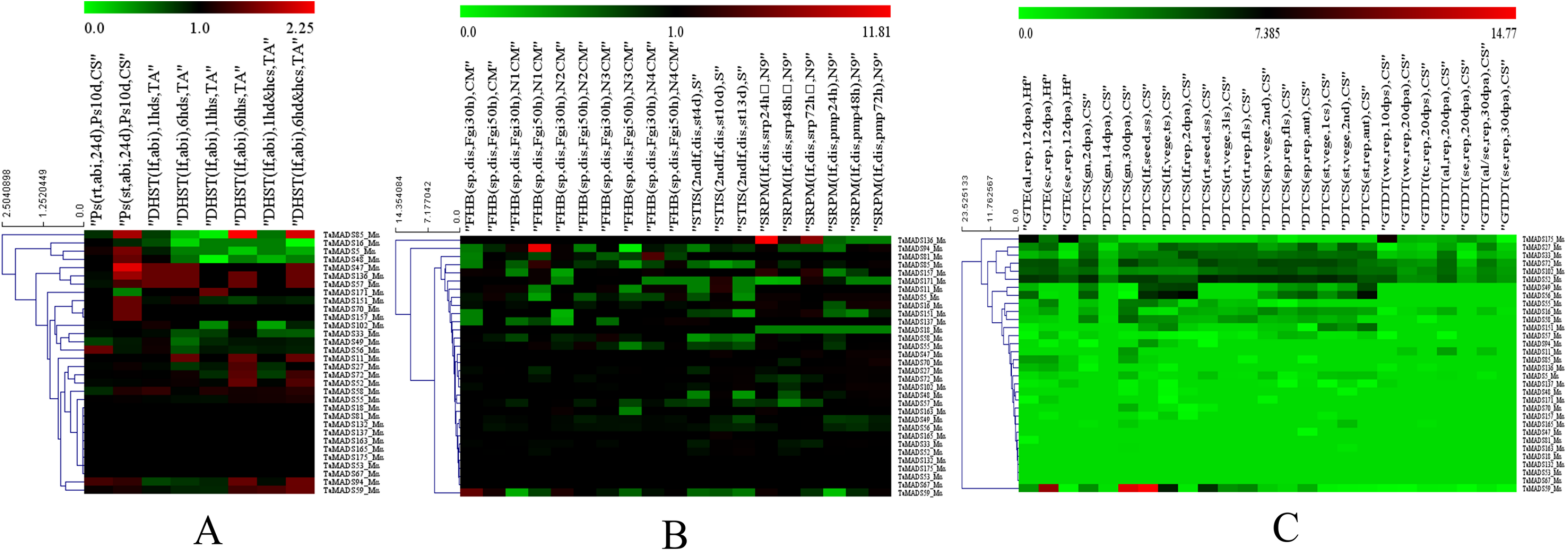
Heatmaps of expression profiles for Mα-type MADS-box genes under stresses. The color scale above represents expression values. **A & B**: abiotic and biotic stresses, respectively. Green and red indicated the expression values decreased and increased, respectively, and black indicated the expression was unregulated. **C**: different tissues and stages, green and red indicated low and high expression values, respectively, and black indicated the average expression values.

Of the 138 MIKC-type genes, 10 and 91 exhibited altered and unaltered gene expression under all four abiotic stresses (phosphorus deficiency, drought, heat and combined drought and heat), respectively (Table 2, S5 Table and S4 Fig). Several genes exhibited substantial differences in expression levels compared to the control. For example, the expression values of *TaMADS121*, *93*, and *21* were seven to four times greater than those of the controls in response to phosphorus deficiency (S5 Table). The expression values of *TaMADS63* and *41* were 1/50 and 1/12, respectively, of those of the controls under heat stress (S5 Table). For the 32 Mα-type genes, 13 and 9 were altered and unaltered in expression, respectively, under all of the stresses (Fig 6 and S5 Table). Interestingly, a majority of the genes showing larger differences between the treatments and controls in expression values were down-regulated. For example, the expression values of *TaMADS16* were 1/6 and 1/66 of those in the controls under heat combined heat and drought treatments, respectively. The expression of only one Mγ-type gene (i.e., *TaMADS164*) was altered under the abiotic stresses and no changed expression values were detected among the Mδ-type genes (Table 2, S5 Table, and S4 Fig).

**Table 2:** Summary of wheat MADS-box gene numbers regulated in response to stresses.

Stresses			Regulated (R) or not (N)	MIKC (138)	Mα (32)	Mγ (5)	Mδ (5)
**Biotic** **stresses**	Fusarium head blight infection		N	49	10	4	4
			R	67	15	1	1
	*Septoria tritici* infection		N	118	10	4	5
			R	16	19	1	0
	Stripe rust pathogen infection		N	117	8	5	5
			R	16	21	0	0
	Powdery mildew pathogen infection		N	119	9	4	5
			R	15	22	1	0
	All the infections		N	41	4	4	4
			R	5	15	1	1
**Abiotic** **stresses**	Phosphate starvation	Root	N	101	16	4	5
			R	37	16	1	0
		Rhoot	N	114	10	4	5
			R	24	22	1	0
	Drought stress	Seedling (1 hour)	N	125	13	4	5
			R	13	19	1	0
		Seedling (6 hours)	N	122	12	4	5
			R	16	18	1	0
	Heat stress	Seedling (1 hour)	N	126	15	3	5
			R	12	17	2	0
		Seedling (6 hours)	N	125	13	3	5
			R	13	19	2	0
	Drought & heat combined stress	Seedling (1 hour)	N	125	16	3	5
			R	13	16	2	0
		Seedling (6 hours)	N	123	12	4	5
			R	15	20	1	0
	All the stresses		N	91	9	3	5
			R	10	13	1	0

#### Biotic stress

Overall, the expression of 41 and 5 MIKC-type genes were unaltered and altered, respectively, in response to all of the four pathogens including *F*. *graminearum*, *S*. *tritici*, stripe rust and powdery mildew (Table 2, S6 Table and S5 Fig). Several genes also showed substantial expression differences between the treatments and the controls. For example, *TaMADS19* increased seven to sixteen times in expression level after being subjected to *S*. *tritici* infection. *TaMADS117* expression was reduced by three to seven times after powdery mildew infection. The expression of 15 and 4 Mα-type genes were, respectively, altered and unaltered in response to all the pathogens (Fig 6 and S6 Table). The expression levels of only one Mγ-type gene (i.e., *TaMADS164*) and one Mδ- type (i.e., *TaMADS83*) gene were altered under the investigated biotic stresses.

#### Vegetative and reproductive development

Overall, 28 MIKC-type genes were expressed in all the investigated tissues including roots, stems, leaves, grains and spikes (Table 2, S7 Table and S6 Fig). The expression of 18 genes could not be detected among any of the five tissues owing to a scarcity of transcripts. The remaining genes were expressed in one to four investigated tissues. The transcripts of 10 Mα-type genes were detected in all of the tissues, while 4 could not be detected at all (Fig 6 and S7 Table). Among Mγ-type genes, only *TaMADS164* was expressed in all the tissues and two genes (*TaMADS66* and *TaMADS68*) were detected in spikes only while two other genes could not be detected in any of the studied tissues. The expression of all five Mδ- type genes were detected in spikes only (S7 Table and S6 Fig).

## Discussion

### Wheat MADS-box genes were likely under-estimated

Despite the identification of 180 MADS-box genes using three different strict methods, there are at least two reasons why our study likely under-estimated the number of genes belonging to the MADS-box family in wheat. Firstly, the available chromosome shotgun sequences do not completely cover each of the three hexaploid wheat sub-genomes [20]. Thus some genes may remain to be identified until the publication of the complete sequence of wheat genome. In this study, no Mβ-type genes were identified by alignments of sequences from wheat and *Arabidopsis*. Further sequence comparisons between either *Brachypodium* or rice and wheat identified five genes (*TaMADS61*, *TaMADS37*, *TaMADS34*, *TaMADS124* and *TaMADS137*) that likely belong to Mβ-type (S7 and S8 Figs). Three of these genes (*TaMADS37*, *TaMADS34* and *TaMADS124*) in wheat were identified as MIKC-type compared to *Arabidopsis* and rice, but they were classified as Mβ-type when comparing to *Brachypodium*. *TaMADS61* was divided into Mβ-type based on both *Brachypodium* and rice, and *TaMADS137* was grouped in Mβ-type (*Brachypodium*) and MIKC-type (rice), respectively. However, *TaMADS61* and *TaMADS137* were classified in Mγ- and Mα-type, respectively based on the classification of the *Arabidopsis* MADS-box gene family (Table 1). The classification of the *Brachypodium* MADS-box gene family was referred to that in rice [8], while the latter was originally conducted based on that in *Arabidopsis* [15]. Thus, it is more reasonable to classify the wheat MADS-box gene family based on the originally classification method in *Arabidopsis* [15]. Phylogenetic analyses of MADS-box genes from both *A*. *tauschii* (S9 and S10 Figs) and *T*. *urartu* (S11 and S12 Figs) with *Arabidopsis* and rice revealed that only *TRIUR3_02276* and *TRIUR3_11471* in *T*. *urartu* possibly fell in the Mβ-type gene classification given the higher bootstrap values (S11 Fig). It is known that rice Mβ-type genes were not identified until the complete sequence were published [15]. Thus, the available more complete wheat gene sequences in the near future will uncover whether Mβ-type genes do exist in wheat or were lost during the evolution.

Secondly, the possibility that gene sequences were not completely assembled also prevented the identification of MADS-box genes. Sequence alignments between the 180 genes identified in this study and 45 reported in a previous study [38] revealed 32 pairs of homologous genes while the remaining 13 could not be matched (Table 1). We further aligned these 13 genes with the whole wheat gene models (v2.1) [20], from which the 180 MADS-box genes were identified, and found that 12 genes could only be matched to partially homologous sequences (percent identities > 98%). For example, the gene *AM502873*.*1* is 1,141 bp in length and matched the gene model, *Traes_6BL_7C6B17284*.*1* which is 774 bp in length with 99% identity. In other words, the available wheat gene model sequences do not appear to be completely assembled yet (likely owing to the lack of domain sequences). This has resulted in the inability to detect some MADS-box genes in the present study despite use of three strict methods. Thus, additional MADS-box genes will be likely detected by future studies.

### Changes in wheat MADS-box gene numbers and chromosomal locations over evolutionary history

The number of wheat MADS-box genes identified in this study exceeds those reported by previous studies (Table 3). One possible explanation is the increased genome size and gene number of wheat relative to other species [17,39,40]. Another possibility is that common wheat is the ultimate result of hybridization among three diploid wheat species and that it has kept most of the MADS-box genes throughout the evolutionary process following the gene duplication event [41–43]. As Mδ-type genes were detected in *Arabidopsis* rather than rice, maize, sorghum and *Brachypodium* (Table 3), it was previously suggested that they are *Arabidopsis*-specific genes [8]. However, the present study also identified five Mδ-type genes in wheat, confirming existence in monocot.

**Table 3:** The classification of MADS-box genes in various species.

Species	Mα	Mβ	Mγ	Mδ	MIKC	Total	Reference
**Common wheat**	32	-	5	5	138	180	this study
**Soybean**	37	14	24	-	88	163	[19]
**Apple**	-	-	-	-	91	147^a^	[17]
**Poplar**	23	12	6	7	57	105	[14]
***Arabidopsis***	25	20	16	6	39	106	[6]
**Rice**	13	9	10	-	43	75	[15]
**Maize**	27	3	2	-	43	75	[16]
**Sorghum**	26	2	2	-	35	65	[16]
***Brachypodium***	9	7	2	-	39	57	[8]
**Cucumber**	5	2	3	3	33	43	[18]

Evolutionary analysis indicated that some members of MADS-box genes could find one or two copies only on one or two chromosomes (Fig 1). Identification of MADS-box genes in wheat and its diploid ancestors, *T*. *urartu* and *A*. *tauschii* suggested that the number of MADS-box genes within a given subgenome was seriously reduced in the transition from diploidy to hexaploidy (for A subgenome, 84 to 57 genes and for B subgenome, 97 to 63 genes). These results further demonstrate that gene loss occurred widely during the formation of hexaploid wheat [44,45].

The fact that the genes belonging to a single group (i.e. different copies of a member of MADS-box) are from different homeologous groups (e.g., *Traes_7AS_CA6E66D75*.*1*, *Traes_7DS_90668ED2B*.*1* and *Traes_6BL_E1793636C*.*1*, Fig 1) indicated that they were most likely involved into inter-chromosomal rearrangements [46,47]. Generally, a high collinearity of MADS-box families has been detected among *T*. *urartu*, *A*. *tauschii* and wheat genomes (Fig 5), suggesting that the evolution of the MADS-box family has been conservative following the formation of hexaploid wheat. However, differences in chromosome locations also exist among several orthologous gene pairs (e.g., *TaMADS8*, *24*, *75* and *124*), indicating that chromosomal rearrangements have occurred throughout the evolution of the MADS-box family in wheat.

### Involvement of MADS-box genes during wheat growth and development

It has been documented that the MADS-box gene family plays key roles in the regulation of flowering time, floral meristems, fruit formations and the development of flower organs and seeds [6,8,15,16,19]. We, here, have identified quite many wheat MADS-box genes that were expressed throughout the investigated tissues and development stages. For example, *TaMADS33*, *52*, *72*, *102*, *120*, and *135* were expressed in roots, stems, leaves, spikes and grains including transfer cells, aleurone layers, starchy endosperms, as well as seed coats during different developmental stages (S7 Table). These results further demonstrated that MADS-box genes have important regulatory functions throughout wheat growth, development and reproductive processes.

The orthologs from different species may play similar roles. However, numerous studies have also reported that numerous orthologs including MADS-box genes from various species likely have different functions as well [8,38,48].The present study found that the expression patterns of a majority of genes were similar to their orthologs in other species such as rice. For example, *TaMADS36* was expressed in all analyzed tissues including roots, stems, leaves, spikes and grains. This pattern was similar to that of its rice ortholog *OsMADS18* [15]. Transcripts of *TaMADS22* and *129* were detected mainly in seeds and spikes, in accordance with their counterparts *OsMADS29* and *58*, respectively [15]. Comparisons also revealed that some orthologs had different expression profiles. For example, *TaMADS114* was expressed in roots, stems, leaves, spikes and grains, resembling the pattern of its rice ortholog, *OsMADS14* [15]. However, this pattern was quite different from its orthologs *BdMADS33* and *AT5G60910*.*1* in *Brachypodium* and *Arabidopsis*, respectively, which were mainly expressed in reproductive organs [6,8]. Another example is that the gene *TaMADS62* was not expressed in seeds but its rice ortholog *OsMADS32* was highly expressed in seeds and during early stages of panicle development [15]. These results and those reported previously offered indications about the possible functional divergence beyond the initial divergence of different species.

The results that some genes exhibited tissue-specific expression patterns have been reported previously [49]. We also detected quite a few of such genes expressed in a single tissue (S7 Table). Interestingly, most of such genes (22/38) in MIKC type were expressed in roots only (Table 2), suggesting that MIKC-type MADS genes could play important roles in development of roots and/or uptake of water and nutrition in roots. The results provided important clues for further gene function research.

Comparing the expression patterns of MADS-box genes from the present study with those reported by Paolacci et al. [38] in which RT-qPCR was employed for expression analysis indicated that most of the characterized genes exhibited consistent expression patterns. For example, *TaMADS31* and *AM502871* were both expressed in all analyzed tissues [38]. These results further strengthen the reliability of conclusions based on transcriptome data analyses in the present study.

In accordance with previous studies [8,15,16], some genes did not appear to have detectable expression as well, possibly for the following reasons as proposed by Zhao et al. [16]: (1) the genes may be pseudogenes that have lost their functions; and (2) these genes may have shown extremely limited temporal and spatial expression patterns and thus the MADS-box genes could be only detected at specific developmental stages or under special conditions. We thus suggest that the MADS-box genes with specific expression should be preferentially selected for cloning and further functional analyses.

### MADS-box gene expressions respond to stresses

Compared to the functions of MADS-box genes in plant growth and development, current understanding of their possible roles in stress responses is rather limited. We thus comprehensively performed expression analyses of MADS-box genes subjected to four abiotic and biotic stresses each to infer their possible roles. Many genes showed substantial expression differences between the stresses and controls, indicating they could be important stress response genes. Indeed, a MADS-box gene has been previously reported to be differentially expressed in response to infection by the stripe rust fungus in wheat, suggesting its potential role in wheat-stripe rust interactions [25]. Thus, these genes would be strong functional candidates for latter research.

Our results also revealed four MIKC-type genes (*TaMADS1*, *41*, *120*, and *135*) and 13 Mα-type genes (*TaMADS16*, *27*, *33*, *49*, *52*, *55*, *56*, *58*, *59*, *72*, *8594*, and *102*,) were regulated under all the biotic and abiotic stresses (S5 and S6 Tables). In *Brachypodium*, one (*BdMADS30*, the ortholog of *TaMADS135*) and three (*BdMADS23*, *33*, and *55*) genes were also identified to be down- and up-regulated, respectively, in all three investigated abiotic treatments including under drought stress by PEG 6000,200 mM NaCl and cold stresses [8]. These results indicated that such genes could be involved between the up- and down-stream of the regulation networks that respond to stresses and thus they may be regulated together. However, further experiments are needed to validate their functions. In addition, most of these genes (e.g., *TaMADS1*, *41*, *120*, and *135*) were expressed in all the investigated tissues including roots, stems, leaves, grains and spikes, suggesting their multiple roles.

Intriguingly, it is likely that Mα-type MADS-box genes are prone to be involved in the regulation of stress response based on the high proportions of Mα-type genes that exhibited expression changes in all the biotic (15 out of 32 genes) and abiotic stresses (13 out of 32 genes) relative to the proportion of MIKC-types genes with altered expression (5 and 10 out of 138 genes, respectively, Table 2).

The lack of experiments designed to examine the responses of MADS-box genes to stresses hinders a more detailed comparison of their possible regulation roles in different species using available data. However, the identified genes exhibiting differential expression under a given stress provide references for similar studies and for further functional analyses.

### The favorable reliability of expression data in this study

The development and improvement of next-generation sequencing technology has enabled the wide use of RNA-seq in various studies across many non-model organisms. The accuracy of RNA-seq results have also been further validated by RT-qPCR in many of these studies [19,50]. Additionally, the well-known public transcriptome database expVIP, which we used a data source, contains RNA-seq data that has been validated by RT-qPCR[37]. Previously, we retrieved RNA-seq results from another widely used transcriptome database, WheatExp[51] to successfully validate the obtained expression values from expVIP[48]. The overall credibility of RNA-seq results justified the use of processed expression values from expVIP in the present study. Additionally, the expression patterns of a majority of genes in this study were in accordance with those of previous studies as discussed above, further supporting the reliability of the retrieved expression data.

## Supporting information

S1 Fig Phylogenetic relationships of the 180 wheat MADS-box genes. The genes on different chromosomes but from a same homeologous group (e.g. *Traes_1AL_5F5A87122*.*1*, *Traes_1BL_B44C0D37C*.*1*, and *Traes_1DL_6DA0DFC5B*.*1*) were represented by a circle filled with red; For the groups where two of the three genes were on different chromosomes from a given homeologous group but the other one was on a chromosome belonging to a different homeologous group (e.g. *Traes_7AS_CA6E66D75*.*1*, *Traes_7DS_90668ED2B*.*1* and *Traes_6BL_E1793636C*.*1*), they were represented by a black circle. The genes represented by a circle filled with black were from a same homeologous group (e.g. *Traes_1AL_5F5A87122*.*1* and *Traes_1DL_D25CDC57D*.*1*). The genes represented by a triangle filled with black were from a single chromosome (e.g. *Traes_4DS_A28BC582A*.*1* and *Traes_4DL_964466BEC*.*1*). The genes represented by a square filled with black were from different homeologous groups (e.g. *Traes_1BS_1202C8C0D*.*1* and *Traes_3DS_51A589227*.*1*). A single gene was represented by a red circle.
(TIF)

S2 Fig Thirty-three pairs of orthologs in wheat and *T*. *urartu*, represented by a circle filled with black, shown in S4 Table. (TIF)

S3 Fig Thirty-two pairs of orthologs in wheat and *A*. *tauschii*, represented by a circle filled with black, shown in S4 Table. (TIF)

S4 Fig **Heatmaps of expression profiles for MADS-box genes (A, B, and D for MIKC, Mγ, and Mδ-type genes, respectively) under abiotic stresses.** Green and red indicated the expression values decreased and increased, respectively, and black indicated the expression was unregulated.
(TIF)

S5 Fig **Heatmaps of expression profiles for MADS-box genes (A, B, and D for MIKC, Mγ, and Mδ-type genes, respectively) under biotic stresses.** Green and red indicated the expression values decreased and increased, respectively, and black indicated the expression was unregulated.
(TIF)

S6 Fig **Heatmaps of expression profiles for MADS-box genes (A, B, and D for MIKC, Mγ, and Mδ-type genes, respectively) in different tissues and stages.** Green and red indicated low and high expression values, respectively, and black indicated the average expression values.
(TIF)

S7 Fig Phylogenetic relationship of MADS-box proteins between *Brachypodium* and wheat. The genes with yellow background were predicted to belong to Mβ-type.
(TIF)

S8 Fig Phylogenetic relationship of MADS-box proteins between rice and wheat. The genes with yellow background were predicted to belong to Mβ-type.
(TIF)

S9 Fig Phylogenetic relationship of MADS-box proteins between *A*. *tauschii* and *A*. *thaliana*. The genes with yellow background belong to Mβ-type.
(TIF)

S10 Fig Phylogenetic relationship of MADS-box proteins between *A*. *tauschii* and rice. The genes with yellow background belong to Mβ-type.
(TIF)

S11 Fig Phylogenetic relationship of MADS-box proteins between *T*. *urartu* and *A*. *thaliana*. The genes with yellow background belong to Mβ-type.
(TIF)

S12 Fig Phylogenetic relationship of MADS-box proteins between *T*. *urartu* and rice. The genes with yellow background belong to Mβ-type.
(TIF)

S1 Table Classification information of MADS-box genes in *A*. *thaliana*. (XLSX)

S2 Table The details of the materials and treatments for the retrieved expression values (extracted from http://www.wheat-expression.com/). (XLSX)

S3 Table Sequence and length of motifs identified from wheat MADS-box proteins using MEME motif search tool (AA, amino acids). (XLSX)

S4 Table Wheat orthologs of MADS-box genes in *T*. *urartu* and *A*. *tauschii*. (XLSX)

S5 Table Expression of wheat MADS-box genes in response to abiotic stresses. (XLSX)

S6 Table Expression of wheat MADS-box genes in response to biotic stresses. (XLSX)

S7 Table Expression patterns of wheat MADS-box genes. (XLSX)
