# Supplementary figures and images for: Genome-wide identification and analysis of the MADS-box gene family in bread wheat (Triticum aestivum L.)
Source: PLoS One. 2017 Jul 25;12(7):e0181443. doi: 10.1371/journal.pone.0181443 (PMC5526560; doi:10.1371/journal.pone.0181443)

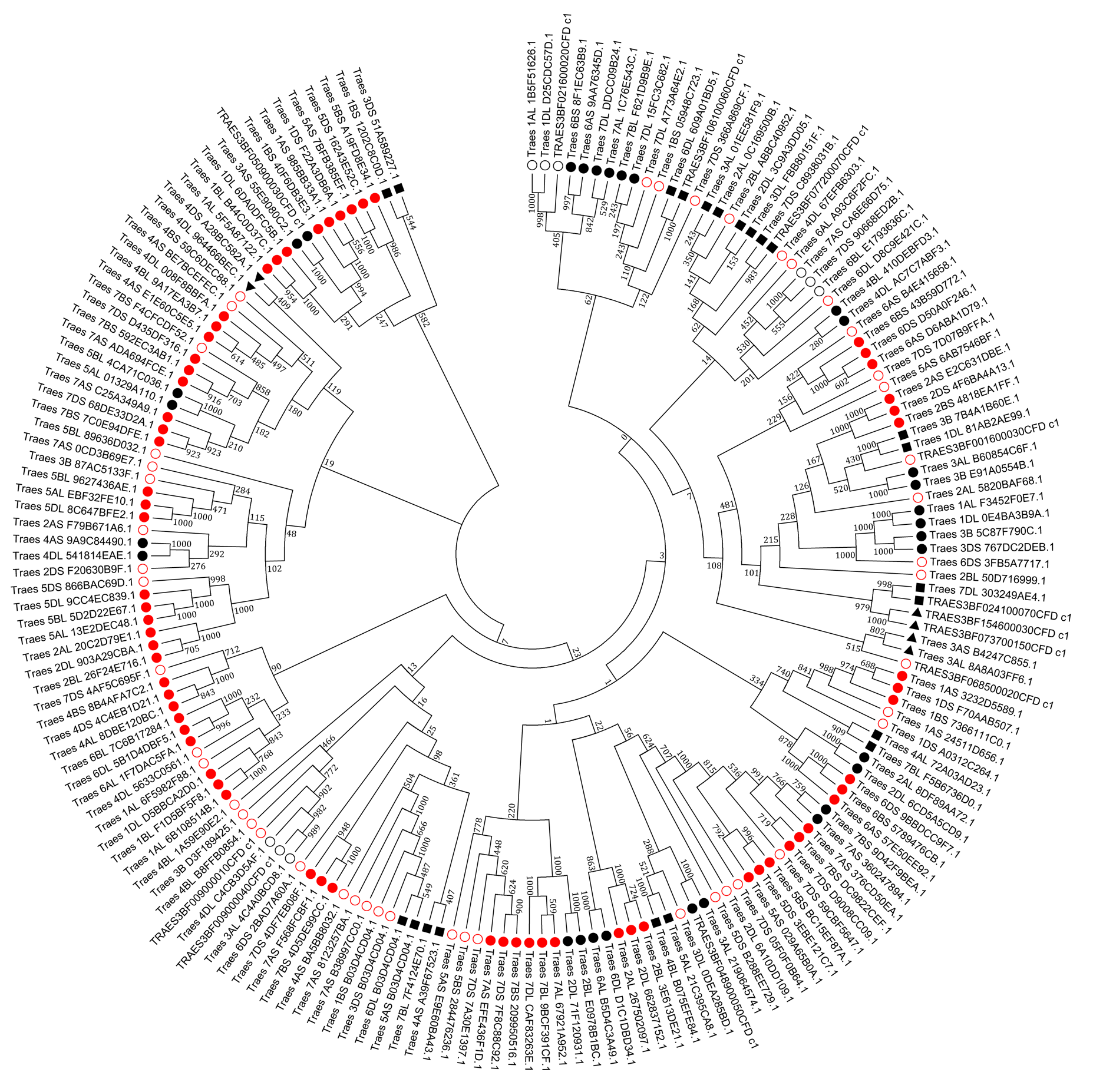

Supplement: S1 Fig — The genes on different chromosomes but from a same homeologous group (e.g. Traes_1AL_5F5A87122.1, Traes_1BL_B44C0D37C.1, and Traes_1DL_6DA0DFC5B.1) were represented by a circle filled with red; For the groups where two of the three genes were on different chromosomes from a given homeologous group but the other one was on a chromosome belonging to a different homeologous group (e.g. Traes_7AS_CA6E66D75.1, Traes_7DS_90668ED2B.1 and Traes_6BL_E1793636C.1), they were represented by a black circle. The genes represented by a circle filled with black were from a same homeologous group (e.g. Traes_1AL_5F5A87122.1 and Traes_1DL_D25CDC57D.1). The genes represented by a triangle filled with black were from a single chromosome (e.g. Traes_4DS_A28BC582A.1 and Traes_4DL_964466BEC.1). The genes represented by a square filled with black were from different homeologous groups (e.g. Traes_1BS_1202C8C0D.1 and Traes_3DS_51A589227.1). A single gene was represented by a red circle. (TIF) [file pone.0181443.s001.tif]

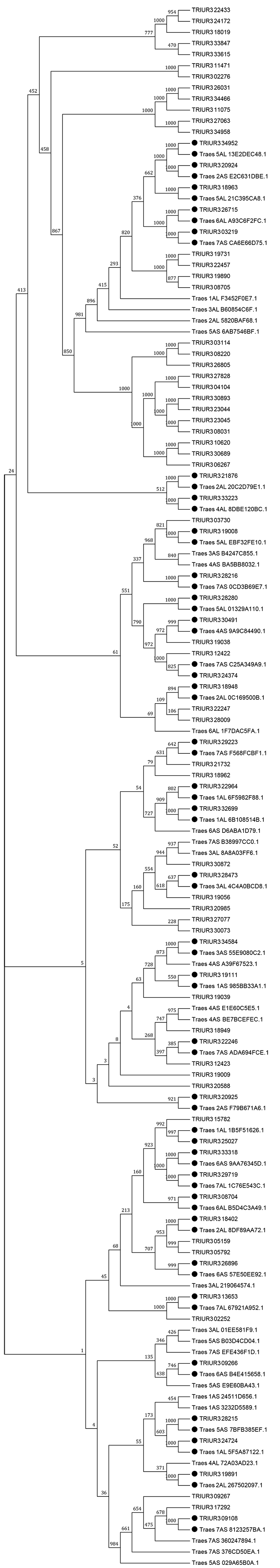

Supplement: S2 Fig — (TIF) [file pone.0181443.s002.tif]

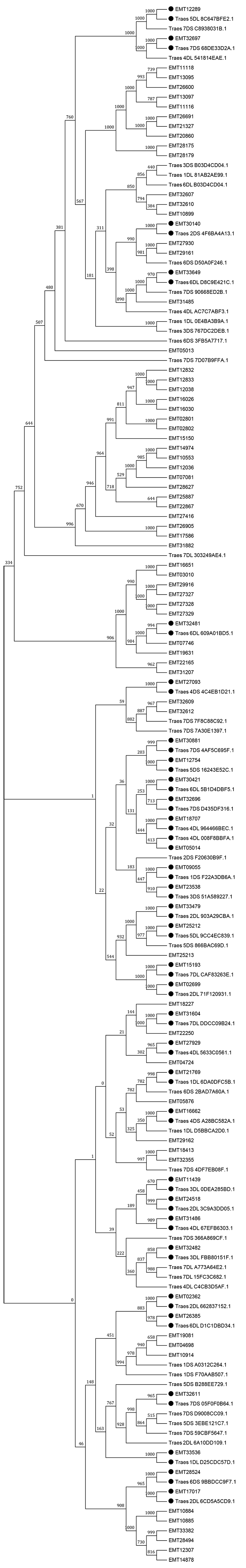

Supplement: S3 Fig — (TIF) [file pone.0181443.s003.tif]

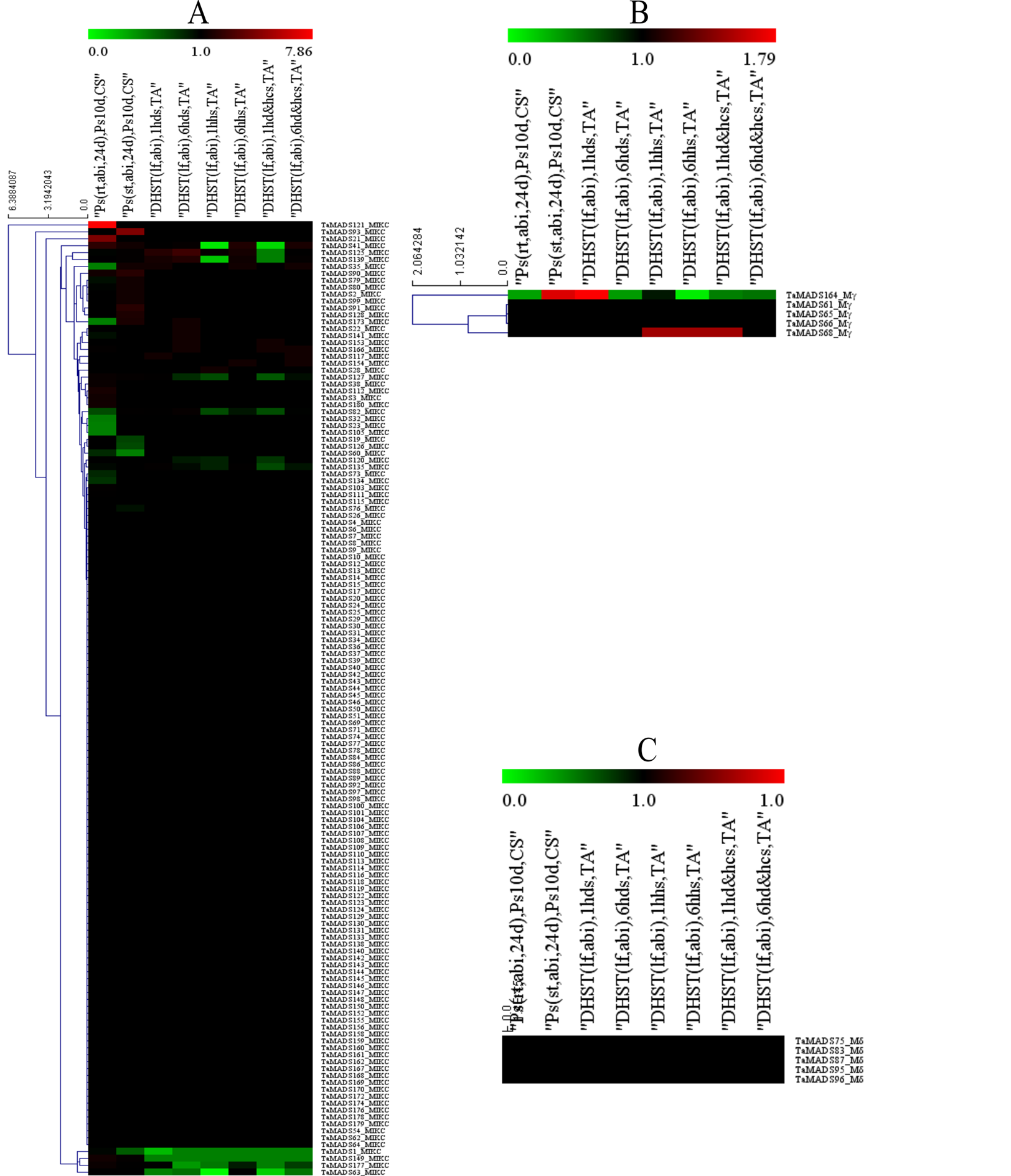

Supplement: S4 Fig — Heatmaps of expression profiles for MADS-box genes (A, B, and D for MIKC, Mγ, and Mδ-type genes, respectively) under abiotic stresses. Green and red indicated the expression values decreased and increased, respectively, and black indicated the expression was unregulated. (TIF) [file pone.0181443.s004.tif]

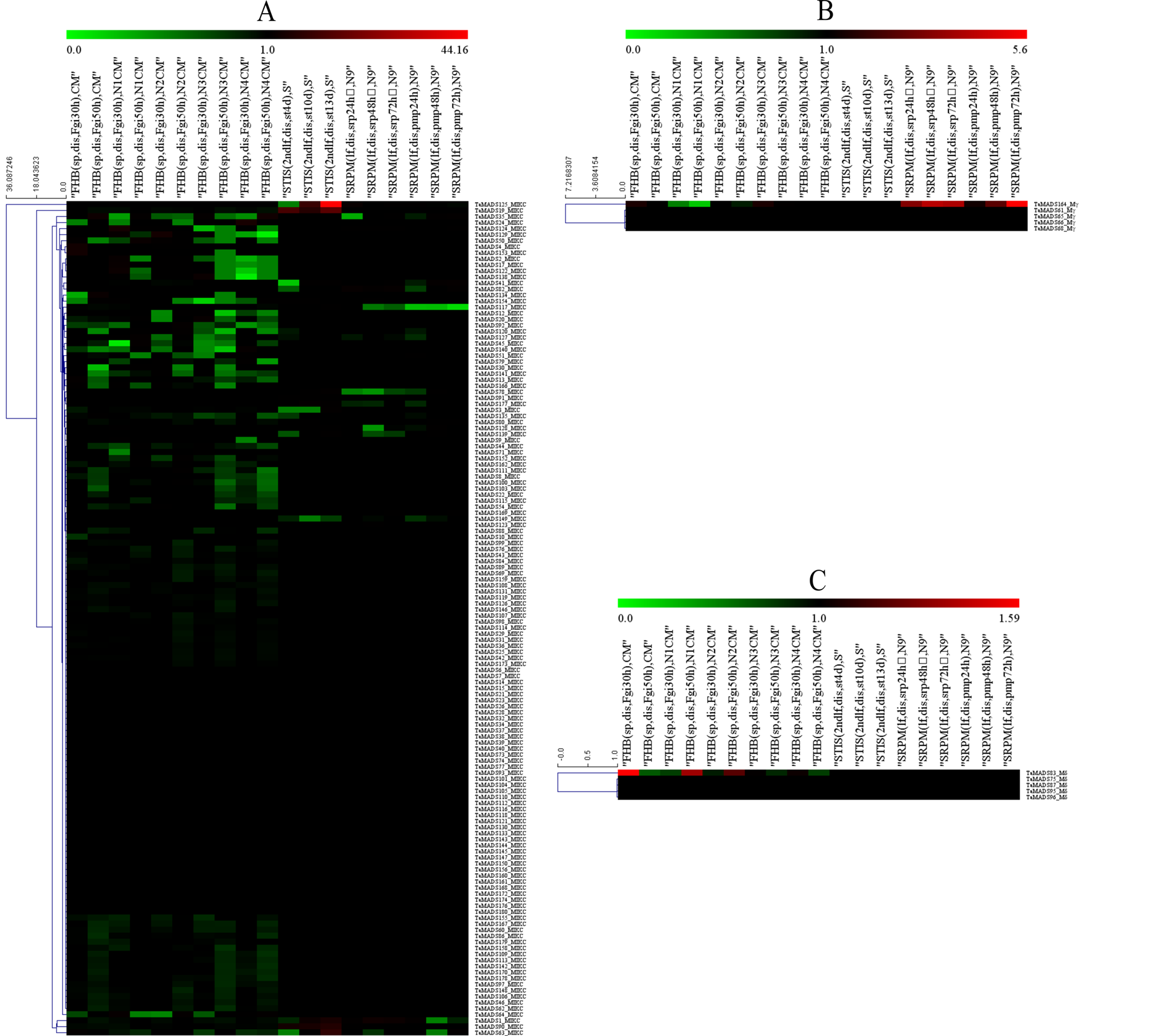

Supplement: S5 Fig — Heatmaps of expression profiles for MADS-box genes (A, B, and D for MIKC, Mγ, and Mδ-type genes, respectively) under biotic stresses. Green and red indicated the expression values decreased and increased, respectively, and black indicated the expression was unregulated. (TIF) [file pone.0181443.s005.tif]

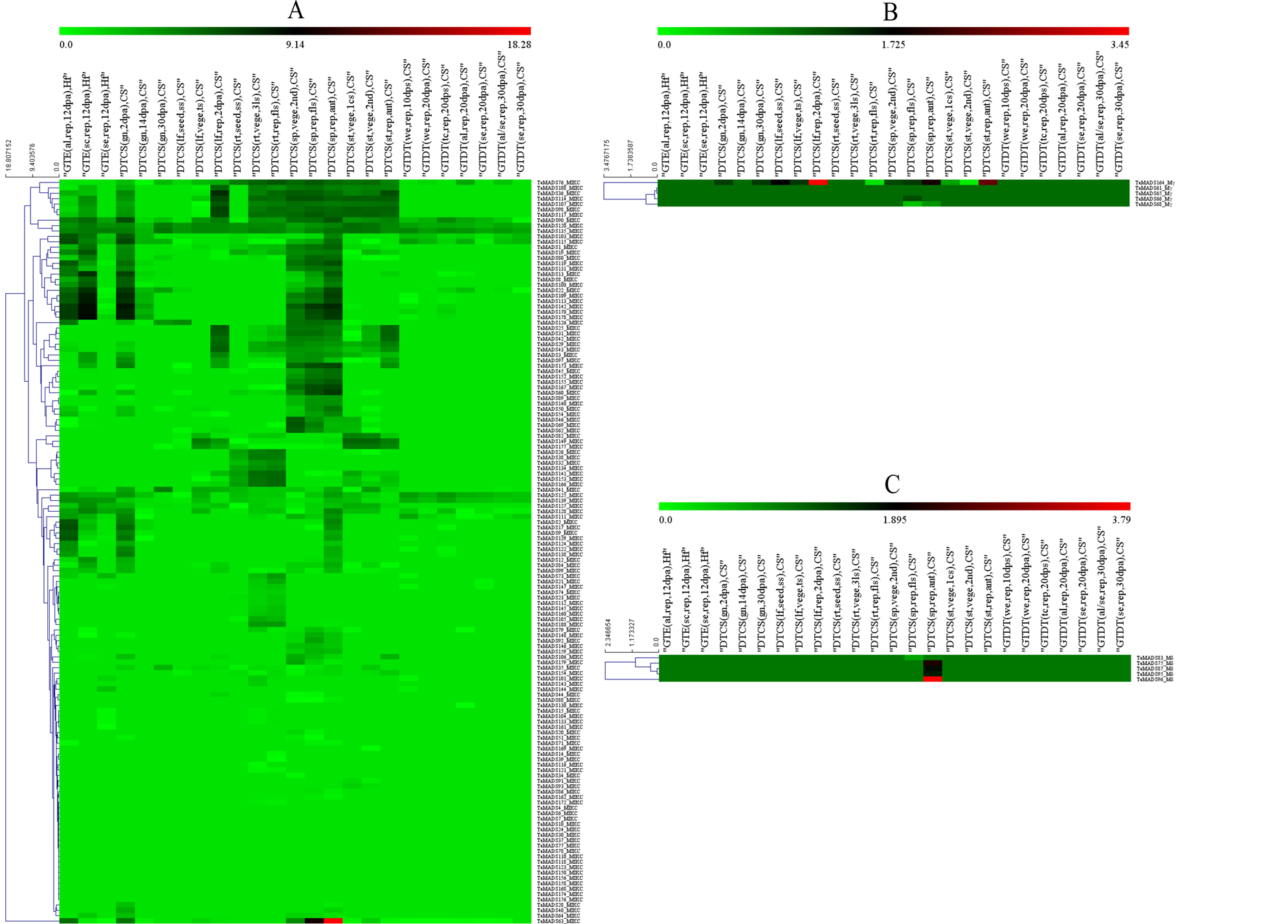

Supplement: S6 Fig — Heatmaps of expression profiles for MADS-box genes (A, B, and D for MIKC, Mγ, and Mδ-type genes, respectively) in different tissues and stages. Green and red indicated low and high expression values, respectively, and black indicated the average expression values. (TIF) [file pone.0181443.s006.tif]

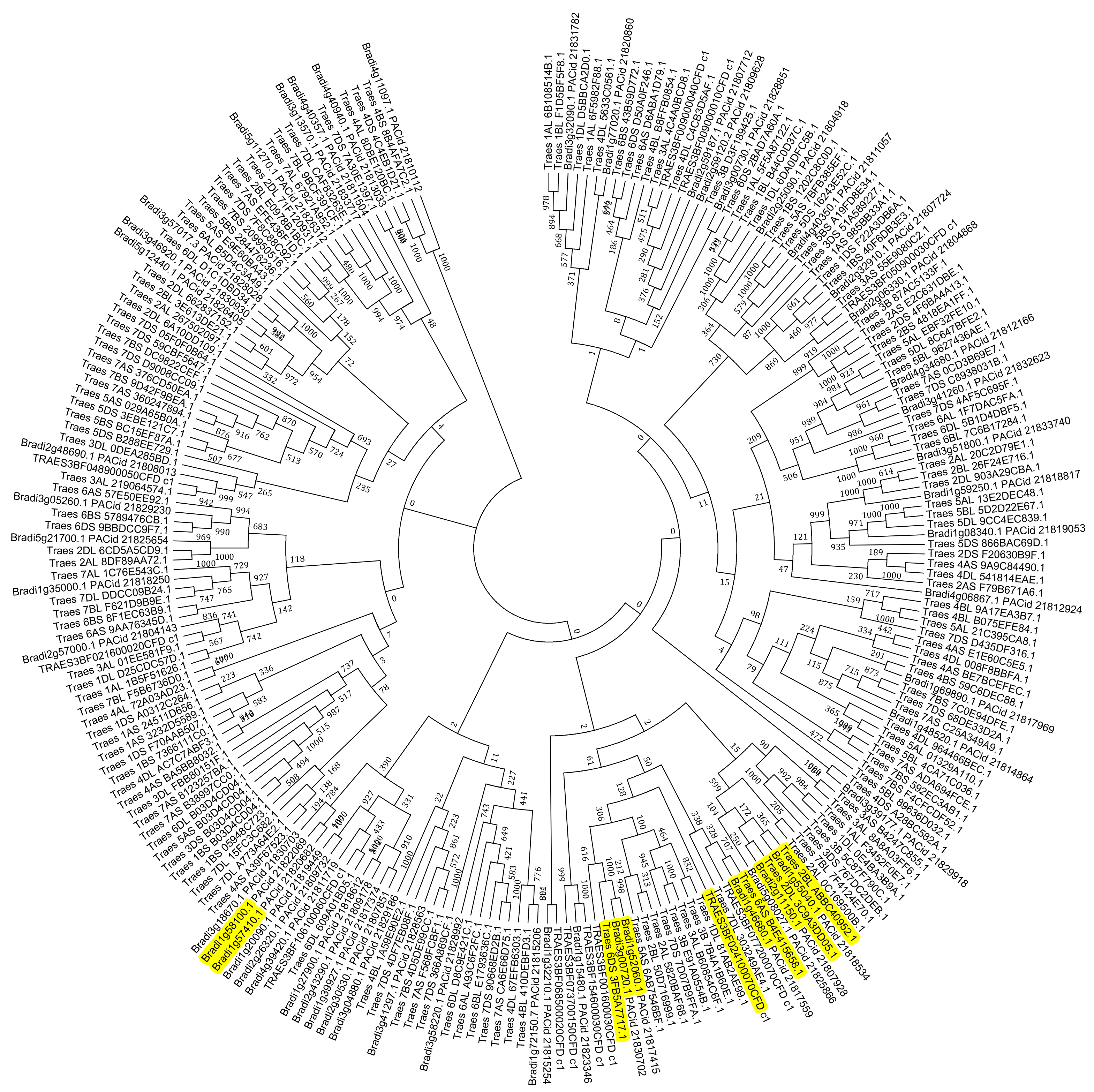

Supplement: S7 Fig — The genes with yellow background were predicted to belong to Mβ-type. (TIF) [file pone.0181443.s007.tif]

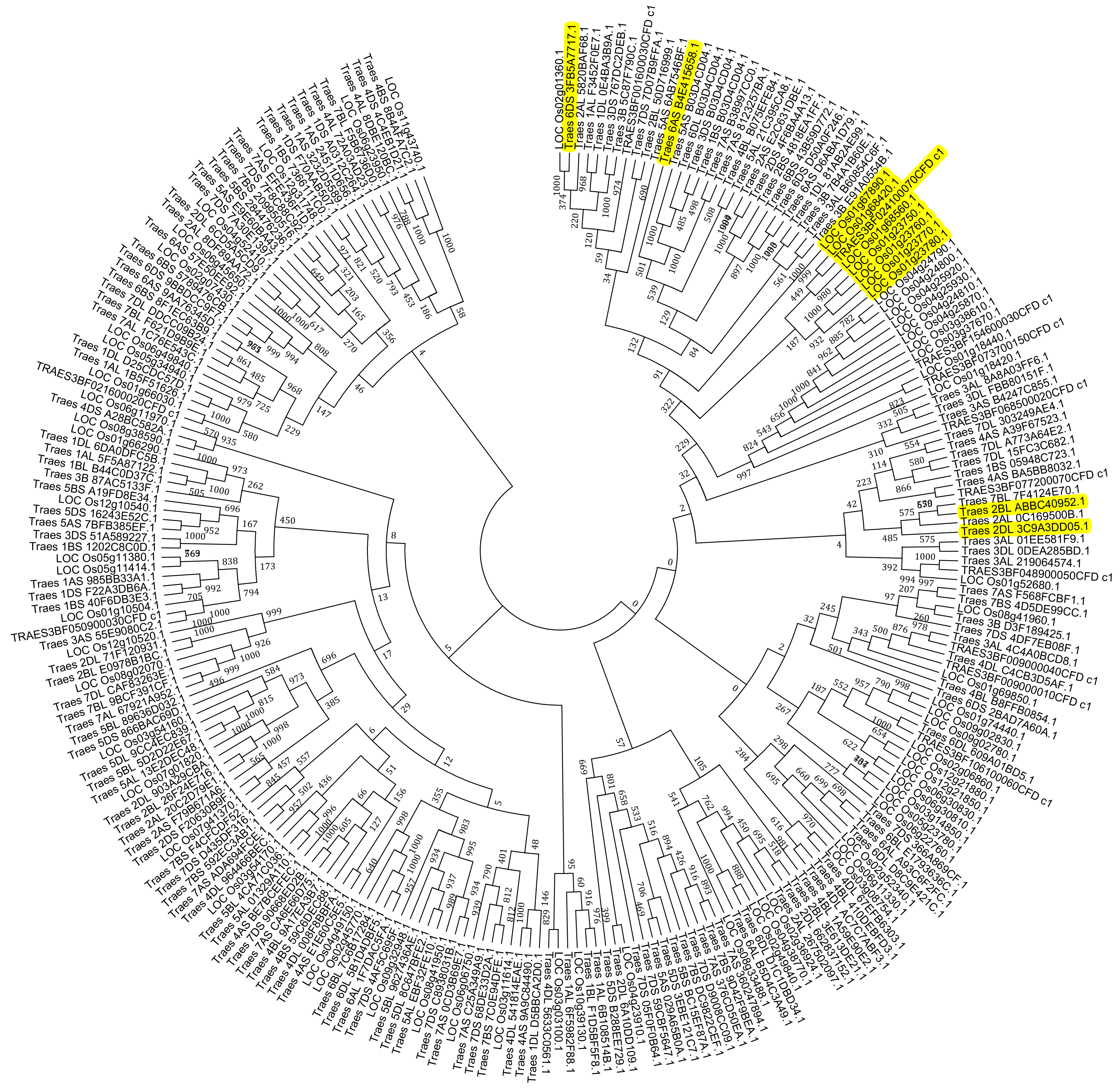

Supplement: S8 Fig — The genes with yellow background were predicted to belong to Mβ-type. (TIF) [file pone.0181443.s008.tif]

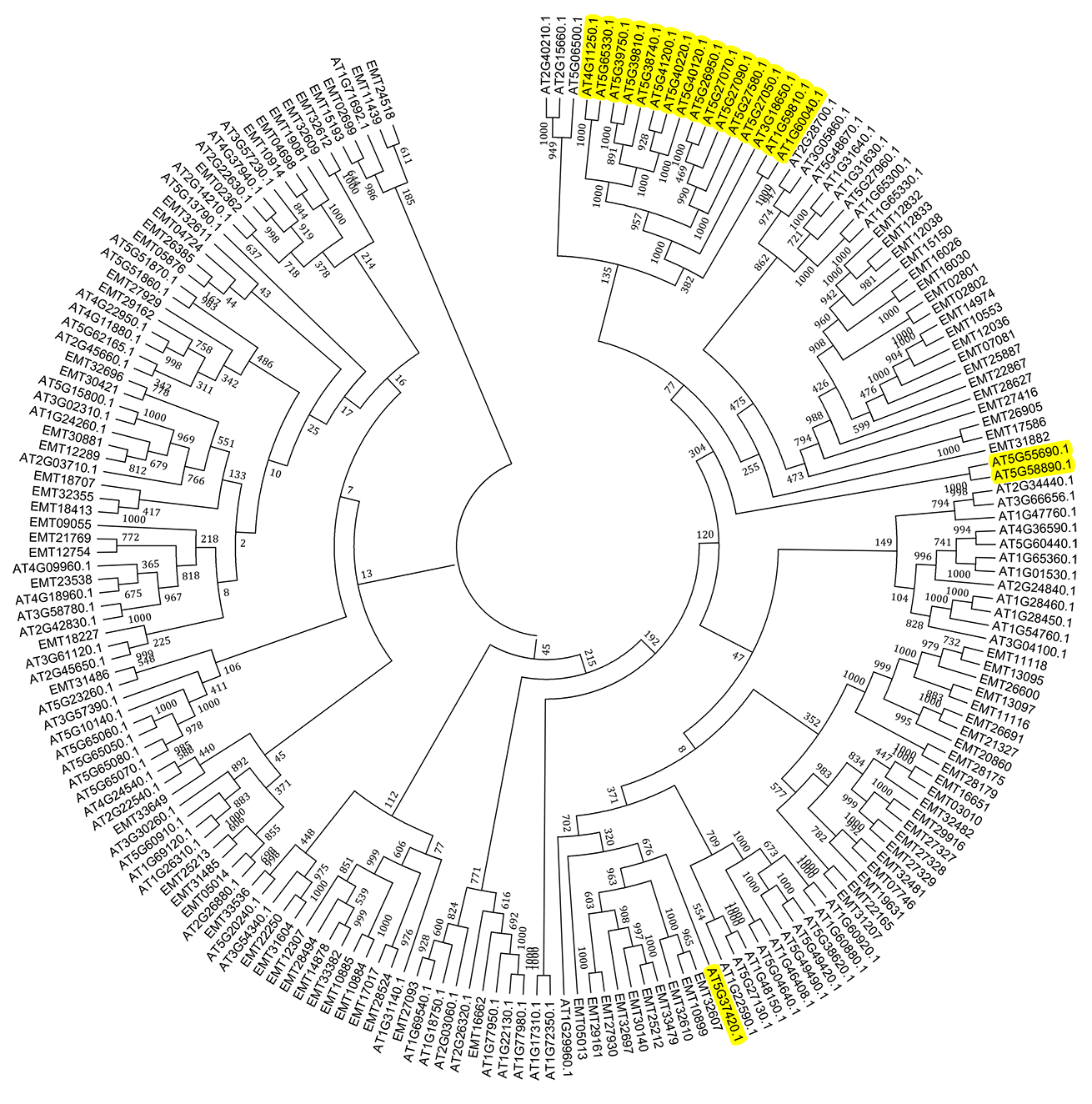

Supplement: S9 Fig — The genes with yellow background belong to Mβ-type. (TIF) [file pone.0181443.s009.tif]

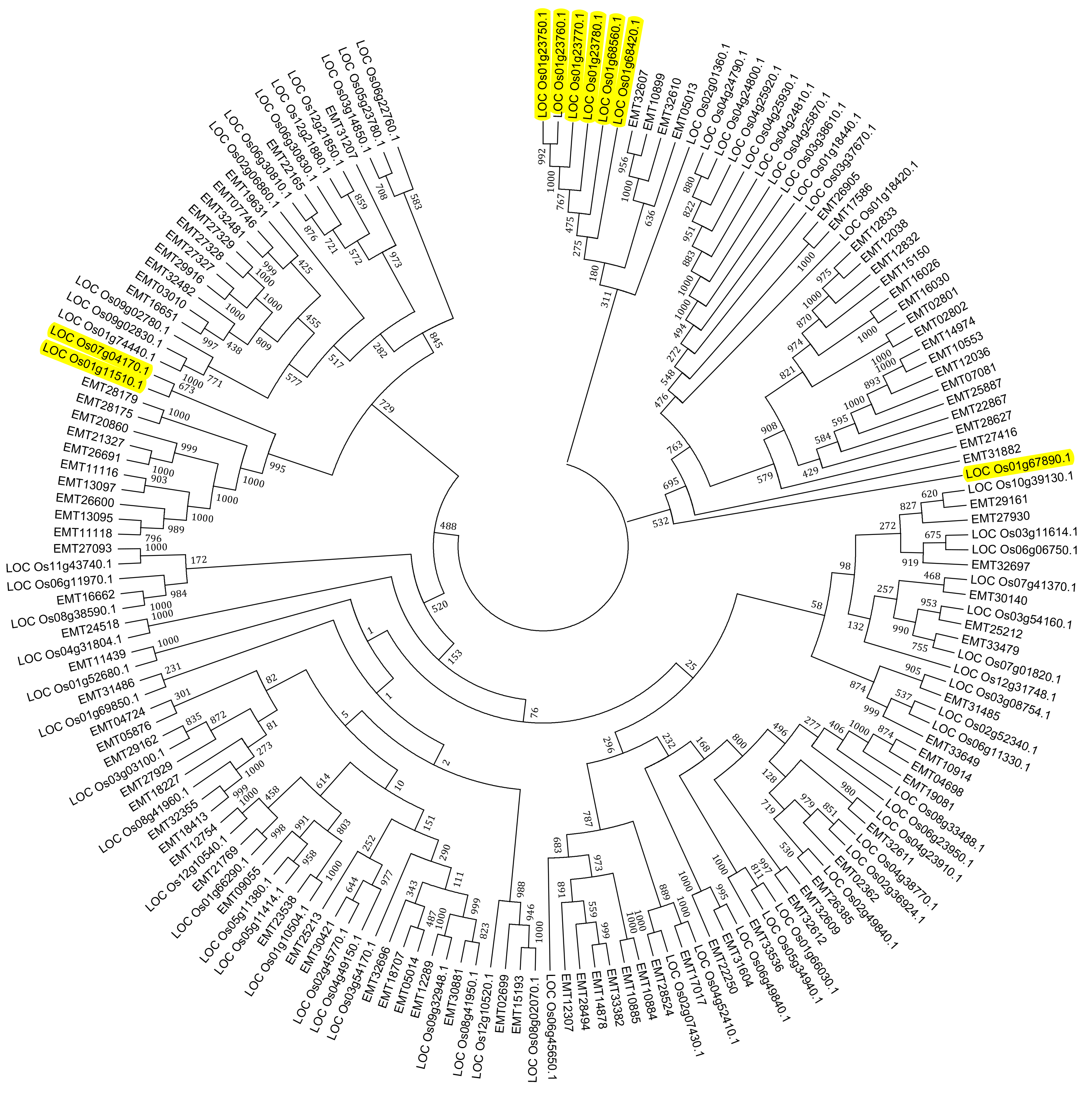

Supplement: S10 Fig — The genes with yellow background belong to Mβ-type. (TIF) [file pone.0181443.s010.tif]

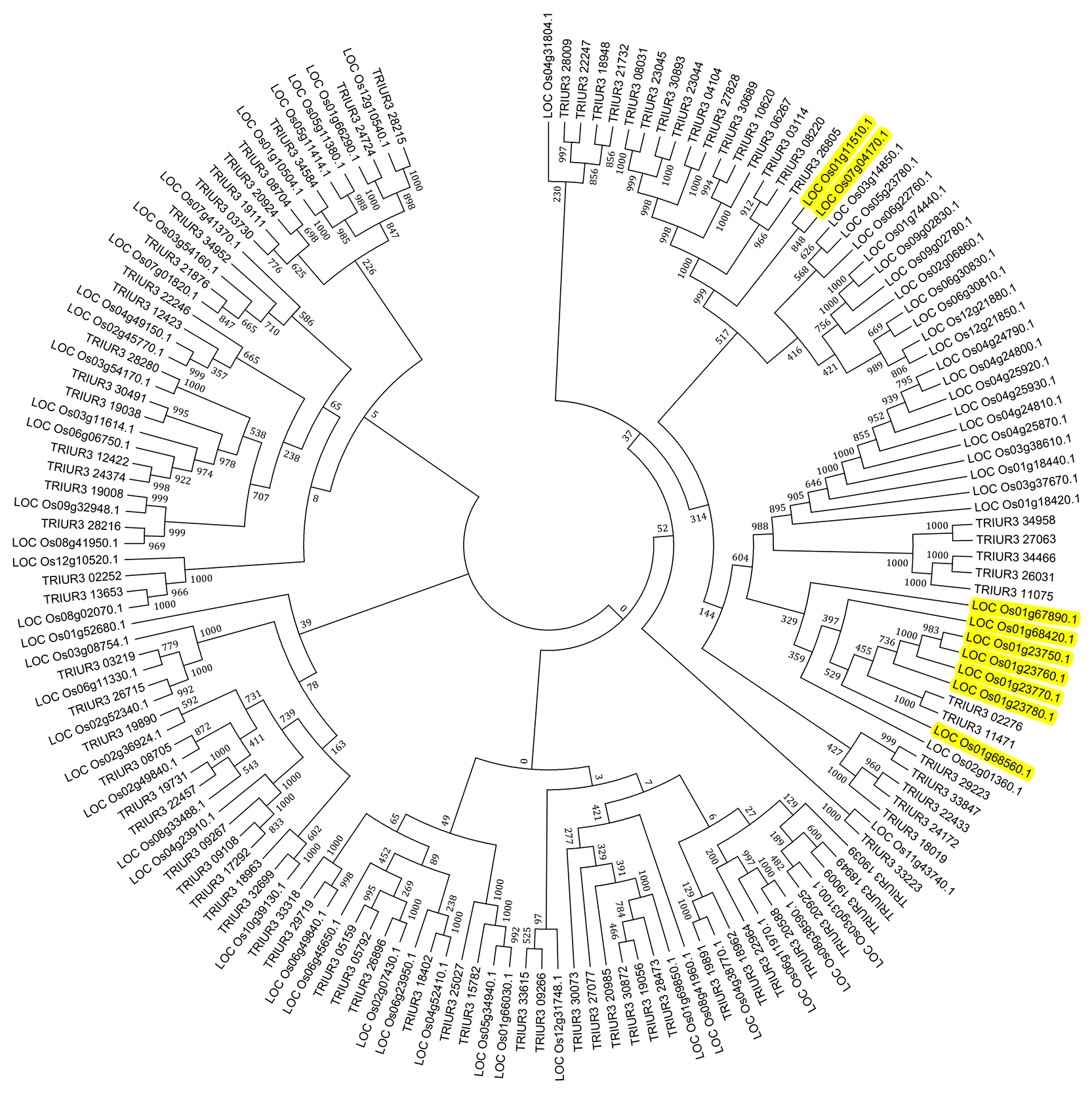

Supplement: S11 Fig — The genes with yellow background belong to Mβ-type. (TIF) [file pone.0181443.s011.tif]

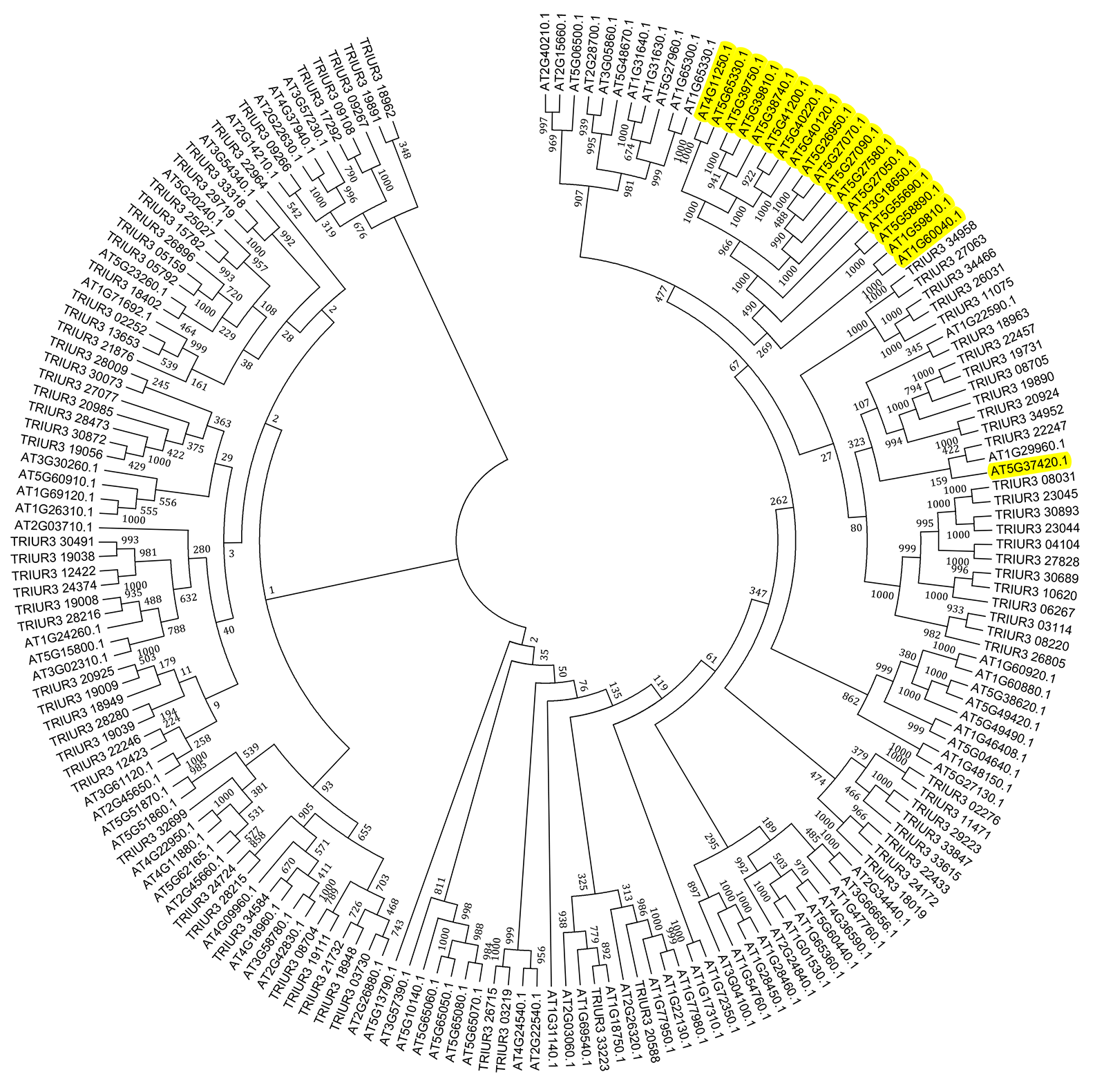

Supplement: S12 Fig — The genes with yellow background belong to Mβ-type. (TIF) [file pone.0181443.s012.tif]
